# Pseudobulbiferamides: Plasmid-Encoded Ureidopeptide Natural Products with Biosynthetic Gene Clusters Shared Among Marine Bacteria of Different Genera

**DOI:** 10.1021/acs.jnatprod.3c00595

**Published:** 2023-09-15

**Authors:** Weimao Zhong, Nicole Aiosa, Jessica M. Deutsch, Neha Garg, Vinayak Agarwal

**Affiliations:** † School of Chemistry and Biochemistry, Georgia Institute of Technology, Atlanta, Georgia 30332, United States; ‡ Center for Microbial Dynamics and Infection, Georgia Institute of Technology, Atlanta, Georgia 30332, United States; § School of Biological Sciences, Georgia Institute of Technology, Atlanta, Georgia 30332, United States

## Abstract

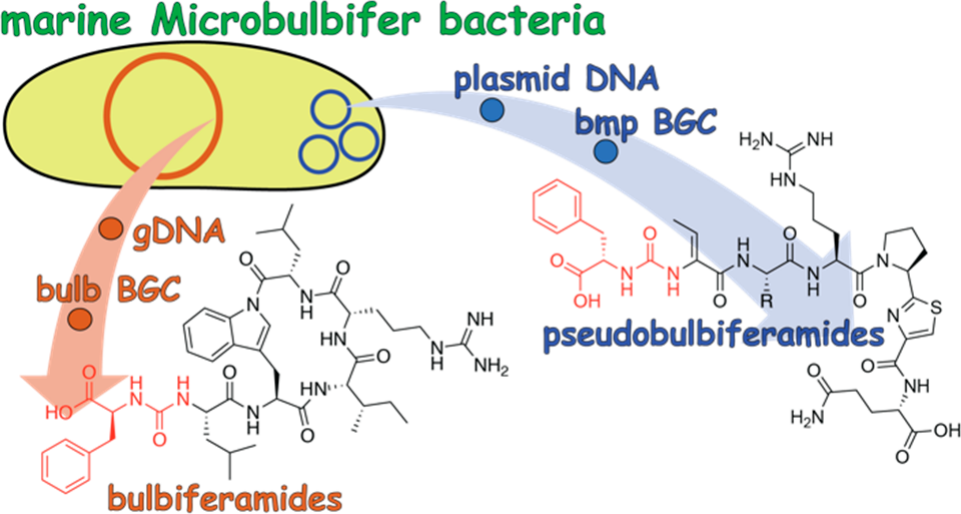

Ureidopeptidic natural products possess a wide variety of favorable pharmacological properties. In addition, they have been shown to mediate core physiological functions in producer bacteria. Here, we report that similar ureidopeptidic natural products with conserved biosynthetic gene clusters are produced by different bacterial genera that coinhabit marine invertebrate microbiomes. We demonstrate that a *Microbulbifer* strain isolated from a marine sponge can produce two different classes of ureidopeptide natural products encoded by two different biosynthetic gene clusters that are positioned on the bacterial chromosome and on a plasmid. The plasmid encoded ureidopeptide natural products, which we term the pseudobulbiferamides (**5**–**8**), resemble the ureidopeptide natural products produced by *Pseudovibrio*, a different marine bacterial genus that is likewise present in marine sponge commensal microbiomes. Using imaging mass spectrometry, we find that the two classes of *Microbulbifer*-derived ureidopeptides occupy different physical spaces relative to the bacterial colony, perhaps implying different roles for these two compound classes in *Microbulbifer* physiology and environmental interactions.

Natural products that play important roles in the physiology of the producer organisms and in mediating their interactions with the environment are widely conserved. Plants produce cyanogenic glycosides that deter herbivory.^1^ Soil-dwelling bacteria produce odoriferous sesquiterpenes to attract anthropods for spore dispersal,^2^ and natural product-mediated mutualistic and antagonistic interactions in entomology are likewise well-established.^3,4^ Gram-negative proteobacteria produce *N*-acyl homoserine lactones to sense and respond to quorum.^5^ In the marine realm, numerous marine algae biosynthesize halomethanes to ward off surface colonization by epiphytic bacteria.^6^ Marine bacteria produce chemical cues that induce settlement of coral larvae.^7^ The genetic potential to produce these natural products is likewise widespread. While the pharmacological activity of natural products often motivates their discovery, the true function of natural products is to fulfill these organismal and ecological roles.

Recently, Berlinck and Eustáquio reported the discovery of ureidopeptidic pseudovibriamide natural products from *Pseudovibrio* bacteria.^8^*Pseudovibrio* are marine Proteobacteria isolated from microbiomes of marine invertebrates such as sponges, tunicates, and corals.^9^ The pseudovibriamide natural products mediate swarming in *Pseudovibrio*, that is, the multicellular ensemble flagellar motion across solid surfaces.^10^ The biosynthetic gene cluster (BGC) responsible for production of pseudovibriamides was found to be positioned on a plasmid. Supporting the primal role of pseudovibriamides in *Pseudovibrio* physiology, the plasmid-encoded pseudovibriamide BGC was found to be widely distributed among *Pseudovibrio* genomes. In this study, we demonstrate that pseudovibriamide-like ureidopeptidic natural products, and highly similar plasmid-encoded BGCs, are shared among not only *Pseudovibrio* spp., but with other bacterial genera in marine invertebrate microbiomes.

The *Pseudovibrio* and *Microbulbifer* genera coinhabit the commensal microbiomes of marine sponges.^11,12^ We, among others, have previously reported the discovery of bulbiferamides **1**–**4** from marine *Microbulbifer* spp. bacteria isolated from sponges *Aplysina fulva* and *Aiolochroia crassa* that were collected in the Florida Keys (Figure 1A).^13,14^ The mass spectrometric fragmentation spectra (MS/MS spectra) for **1**–**4** demonstrated 165 and 26 Da neutral losses characteristic of the Phe residue connected via the ureidopeptide bond. In extracts of the bacterium *Microbulbifer* sp. MKSA007 that was isolated from the sponge *Smenospongia aurea* also collected in the Florida Keys,^11^ we detected metabolites demonstrating similar features in their fragmentation spectra (Figure 1B). *S. aurea*, *A. fulva*, and *A. crassa* are high microbial abundance sponges that are ubiquitous on shallow reefs in the Florida Keys.^15^ However, structures of the *Microbulbifer* sp. MKSA007-derived metabolites could not be discerned based on progression of proteinogenic amino acids.^13,16^ These metabolites were then isolated from liquid culture extracts of *Microbulbifer* sp. MKSA007 and their structure elucidation pursued using spectroscopic and degradation experiments. Given their structural similarity to pseudovibriamides (*vide infra*), we have named these molecules pseudobulbiferamides (Figure 1C).

**Figure 1:**
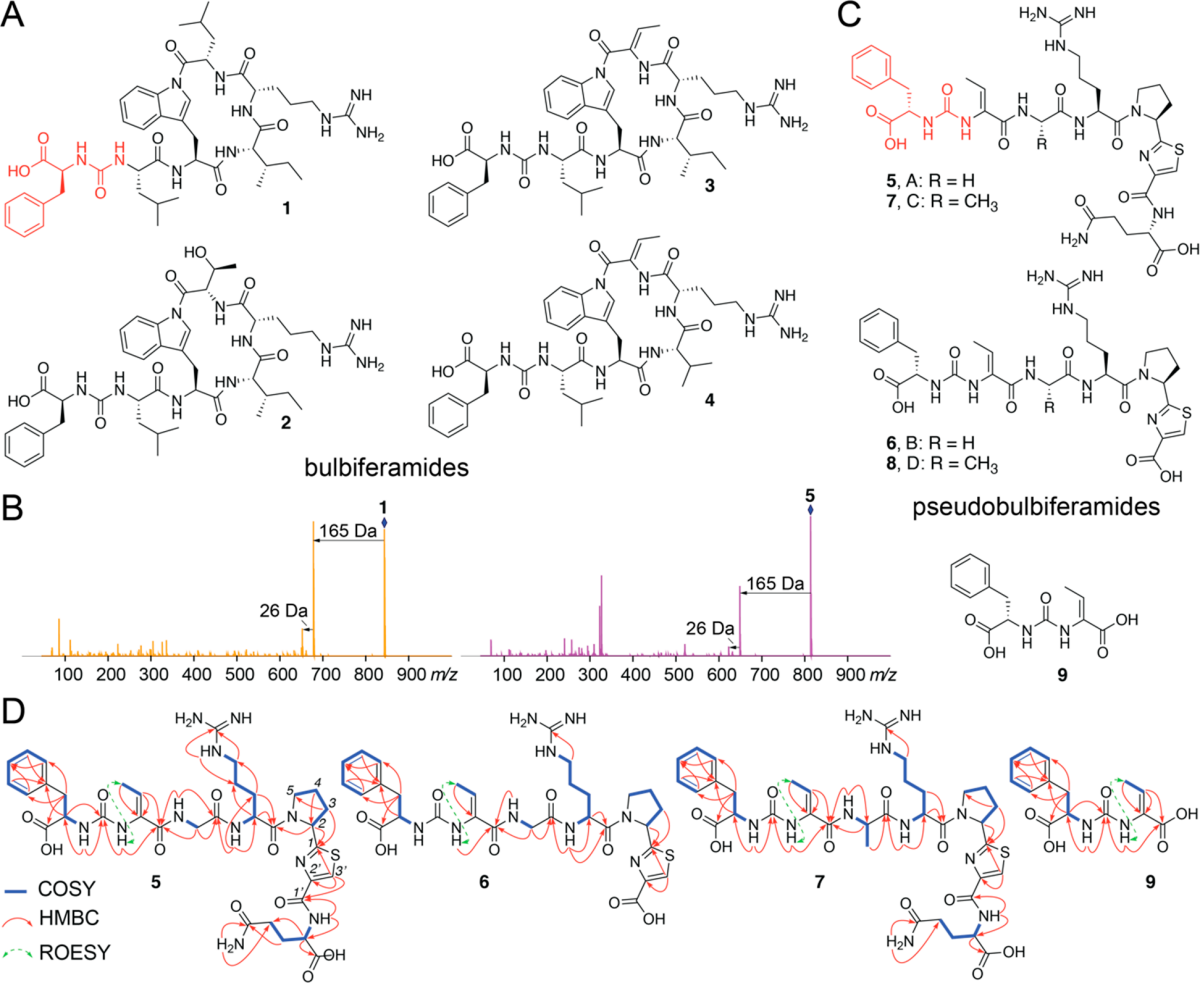
(A) Bulbiferamides isolated from marine *Microbulbifer* sp. bacteria. The Phe residue and the ureido linkage are highlighted in red. (B) MS/MS fragmentation spectra for bulbiferamide A (**1**, left) and pseudobulbiferamide A (**5**, right), demonstrating the conserved 165 and 26 Da neutral losses. Parent ions are marked by the blue diamonds. (C) Structures of pseudobulbiferamides A–D (**5**–**8**) and the shunt metabolite **9** described in this study. (D) COSY, HMBC, and selected ROESY correlations for **5**–**7**, **9**.

Pseudobulbiferamide A (**5**) was isolated as a yellow oil with the molecular formula C_35_H_47_N_11_O_10_S, indicating 18 degrees of unsaturation. The ^1^H NMR spectrum revealed amide proton signals (δ_H_ 6.54–8.44 Table 1) and amino acid α-proton signals (δ_H_ 3.63–5.33), which were suggestive of its peptidic nature. This inference was supported by the observation of amide carbonyl carbon signals (δ_C_ 160.5–173.6) and amino acid Cα carbon signals (δ_C_ 42.3–58.3) in the ^13^C NMR spectra. Analysis of the 2D NMR spectra, including HSQC, HMBC, and COSY allowed the identification of five proteinogenic amino acid residues Phe, Gly, Arg, Pro, and Gln (Figures 1D and S1–S9). Two additional modified residues were identified. First, a dehydrobutyrine (Dhb) residue was determined to be present by the COSY correlation between methyl group protons (δ_H_ 1.62, d, *J* = 7.0 Hz) and a vinyl proton (δ_H_ 5.89, q, *J* = 7.0 Hz) and HMBC correlations from the above-mentioned vinyl proton and an amide proton (δ_H_ 7.93, s) to a Cα carbon atom (δ_C_ 132.1) and an amino acid main chain carbonyl carbon (δ_C_ 165.6). The configuration of the Cα-Cβ double bond in the Dhb residue was deduced to be *Z* by the ROESY correlation between its amide proton and methyl group.^17^ Second, a thiazole heterocycle (Thz) derived from a Cys residue was elucidated based on HMBC correlations from the Thz H_β_ (δ_H_ 8.17, s) to Thz carbonyl carbon (δ_C_ 160.5), Cα-carbon (δ_C_ 149.1), and Pro carbonyl carbon (δ_C_ 173.6). A hexapeptide partial structure Dhb^2^-Gly^3^-Arg^4^-Pro^5^-Thz^6^-Gln^7^ could then be established based on HMBC correlations between the amino acid amide protons or α-protons and the neighboring carbonyl carbon atoms. A ureido bond was deduced to be linking Phe^1^ and Dhb^2^ based on HMBC correlations from both amide protons of Phe^1^ and Dhb^2^ to a deshielded carbon signal at δ_C_ 155.3, which is a characteristic chemical shift of ureido carbonyl carbons.^18,19^ The planar structure of **5** was thus deduced as a linear heptapeptide Phe^1^-Dhb^2^-Gly^3^-Arg^4^-Pro^5^-Thz^6^-Gln^7^ with a ureido linkage between the Phe^1^ and Dhb^2^ residues. The absolute configurations of all amino acids were determined to be L by Marfey’s analysis (Figures S10–S13). Progressing from **5**, using MS/MS fragmentation spectra, we could discern the structure of pseudobulbiferamide B (**6**), wherein the terminal Gln residue was omitted (Figure S14). Structural assignment of **6** was supported by examination of NMR spectra (Figures S15–S21, Table S1).

**Table 1:** ^13^C (176 MHz) and ^1^H (700 MHz) NMR Chemical Shifts of Compounds **5** and **7** in DMSO-*d*_6_ (*J* in Hz, δ in ppm)

		5		7	
residue	no.	δ_C_, type^a^	δ_H_, (*J*, Hz)^b^	δ_C_, type^a^	δ_H_, (*J*, Hz)^b^
Phe^1^	1	173.1, C		173.2, C	
	2	53.8, CH	4.43, dt (7.4, 6.0)	53.8, CH	4.39, overlap
	3	37.5, CH_2_	3.05, dd (13.7, 5.3)	37.5, CH_2_	3.03, dd (13.7, 5.3)
			2.95, dd (13.7, 7.4)		2.95, dd (13.7, 6.9)
	4	137.0, C		137.1, C	
	5/9	129.5, CH	7.17, d (7.1)	129.4, CH	7.20, d (7.0)
	6/8	128.2, CH	7.28, t (7.3)	128.2, CH	7.29, t (7.6)
	7	126.5, CH	7.22, t (7.3)	126.6, CH	7.35, t (7.3)
	NH		6.54, d (7.6)		6.47, d (7.9)
Ureido	CO	155.3, C		155.1, C	
Dhb^2^	1	165.6, C		164.9, C	
	2	132.1, C		131.7, C	
	3	120.6, CH	5.89, q (7.0)	121.9, CH	5.96, q (7.0)
	4	12.3, CH_3_	1.62, d (7.0)	12.7, CH_3_	1.59, d (7.0)
	NH		7.93, s		7.76, s
Gly^3^	1	169.2, C			
	2	42.3, CH_2_	3.79, overlap		
			3.63, dd (16.7, 5.8)		
	NH		8.08, t (5.9)		
Ala^3^	1			172.3, C	
	2			48.3, CH	4.30, dq (7.2, 6.9)
	3			18.0, CH_3_	1.23, d (7.1)
	NH				7.71, d (7.5)
Arg^4^	1	170.7, C		172.4, C	
	2	50.2, CH	4.55, m	48.3, CH	4.52, m
	3	28.0, CH_2_	1.77, m	28.2, CH_2_	1.76, m
			1.67, m		1.61, overlap
	4	24.9, CH_2_	1.56, m	24.9, CH_2_	1.55, m
			1.52, m		1.52, m
	5	40.4, CH_2_	3.09, m	40.1, CH_2_	3.10, m
	6	156.7, C		156.6, C	
	αNH		8.12, d (7.5)		8.15, d (7.5)
	δNH		7.59, t (5.0)		7.48, br s
	εNH		nd		nd
Pro^5^ -Thz^6^	1	173.6, C		173.6, C	
	2	58.3, CH	5.33, dd, (6.4, 4.9)	58.4, CH	5.32, dd, (8.0, 2.8)
	3	31.4, CH_2_	2.23, m	31.4, CH_2_	2.21, m
	4	24.0, CH_2_	1.99, overlap	24.1, CH_2_	1.97, m
			2.03, m		1.99, m
	5	46.8, CH_2_	3.77, overlap	46.7, CH_2_	3.73, m
	1′	160.5, C		160.4, C	
	2′	149.1, C		149.1, C	
	3′	123.9, CH	8.17, s	124.0, CH	8.18, s
Gln^7^	1	173.2, C		173.3, C	
	2	51.8, CH	4.39, m	51.8, CH	4.39, m
	3	26.4, CH_2_	2.11, m 1.97, overlap	26.3, CH_2_	2.11, m 1.97, overlap
	4	31.4, CH_2_	2.15, t, (6.4)	31.4, CH_2_	2.15, t, (6.4)
	5	173.6, C		173.5, C	
	αNH		8.44, d (7.9)		8.43, d (7.8)
	δNH		7.32, s		7.32, s
			6.78, s		6.78, s

a Recorded at 176 MHz.

b Recorded at 700 MHz. nd: not detected.

Pseudobulbiferamide C (**7**) was isolated as a yellow oil with the molecular formula C_36_H_49_N_11_O_10_S. The ^1^H and ^13^C NMR data of **7** were highly similar to those of **5** (Table 1). Analysis of MS/MS fragmentation and 2D NMR spectra indicated that the Gly residue in **5** was replaced by an Ala residue in **7**, as discerned by the COSY correlation between the Ala methyl protons (δ_H_ 1.23, d, *J* = 7.1 Hz) and the Ala α-proton (δ_H_ 4.30, dq, *J* = 7.2, 6.9 Hz), as well as HMBC correlations from the Ala methyl protons to the Ala amide carbonyl carbon (δ_C_ 172.3) (Figures S22–S31). As before, the configuration of Dhb residue was deduced to be *Z* by a ROESY correlation between its amide proton and methyl group. Thus, the planar structure of **7** was determined as Phe^1^-Dhb^2^-Ala^3^-Arg^4^-Pro^5^-Thz^6^-Gln^7^ with a ureido bond connecting the Phe^1^ and the Dhb^2^ residues (Figure 1D). Marfey’s analysis of **7** allowed assignments of the amino acids as L (Figures S32–S36). Akin to **6**, MS/MS data demonstrated the presence of pseudobulbiferamide D (**8**) wherein the terminal Gln residue was omitted (Figure S37). Molecule **8** was produced in drastically reduced amounts which precluded isolation and NMR spectra acquisition. We also detected the presence of the shunt metabolite **9** comprising of just the Phe and Dhb residues connected via the ureido linkage. Examination of the NMR spectra allowed assignment of the Dhb Cα-Cβ double bond in **9** to be in the *Z* configuration (Figures S38–S44, Table S1). Marfey’s analysis allowed assignment of the Phe residue in **9** as l-Phe (Figure S45).

The structures of the pseudobulbiferamides are similar to the pseudovibriamides, hybrid nonribosomal peptide synthetase/polyketide synthase (NRPS/PKS) derived natural products isolated from extracts of a marine *Pseudovibrio* bacterium, which, akin to *Microbulbifer* sp. MKSA007, was isolated from a marine sponge. Crucially, the Phe^1^ residue in pseudobulbiferamides is replaced with Tyr in pseudovibriamides which distinguishes the two compound families.

The biosynthesis of pseudovibriamides was mapped to the plasmid-encoded *ppp* BGC.^8^ The *ppp* BGC is widespread in marine *Pseudovibrio* strains. Sequencing and assembly of a draft genome of *Microbulbifer* sp. MKSA007 identified a highly similar BGC, which we term the *Microbulbifer*-derived *ppp*-like BGC (*mbp* BGC). Just like the *ppp* BGC, the *mbp* BGC is plasmid encoded (Figure 2A). The presence of the *ppp* and the *mbp* BGCs on plasmids could have functional relevance to the distribution of these BGCs and the corresponding ureidopeptidic natural products in marine invertebrate microbiomes. The organization of the *mbp* BGC wherein genes *mbpA–C* encode a heptamodular NRPS assembly line leads to a biosynthetic proposal for the production of pseudobulbiferamides (Figure 2B). Ureido bond formation, dehydration of the Thr side chain to Dhb and the Cys cyclodehydration to thiazoline is proposed to be catalyzed by the corresponding NRPS condensation domains.^20−23^ An oxidase domain at the C-terminus of the NRPS MbpB is proposed to catalyze the thiazoline to thiazole transformation. Of note is the presence of the *mbpD* gene which encodes two additional NRPS modules followed by a PKS module. The corresponding gene in the *ppp* BGC, *pppD*, extends the pseudovibriamide ureidoheptapeptides with an additional acetate unit and a prolyl dipeptide.^8^ The correspondingly extended congeners were not detected for the pseudobulbiferamides. Genes *mbpE* and *mbpF* encode a phosphopantetheinyl transferase and a thioesterase, respectively. Additional thioesterases are encoded at the C-termini of the MbpC and MbpD polypeptides. Putative transporters are encoded by *mbpG* and *mbpL*. The catalytic roles if any, of the unannotated open reading frames MbpH–J, and the α-ketoglutarate-dependent oxidase MbpK in the biosynthesis of pseudobulbiferamides are not apparent.

**Figure 2:**
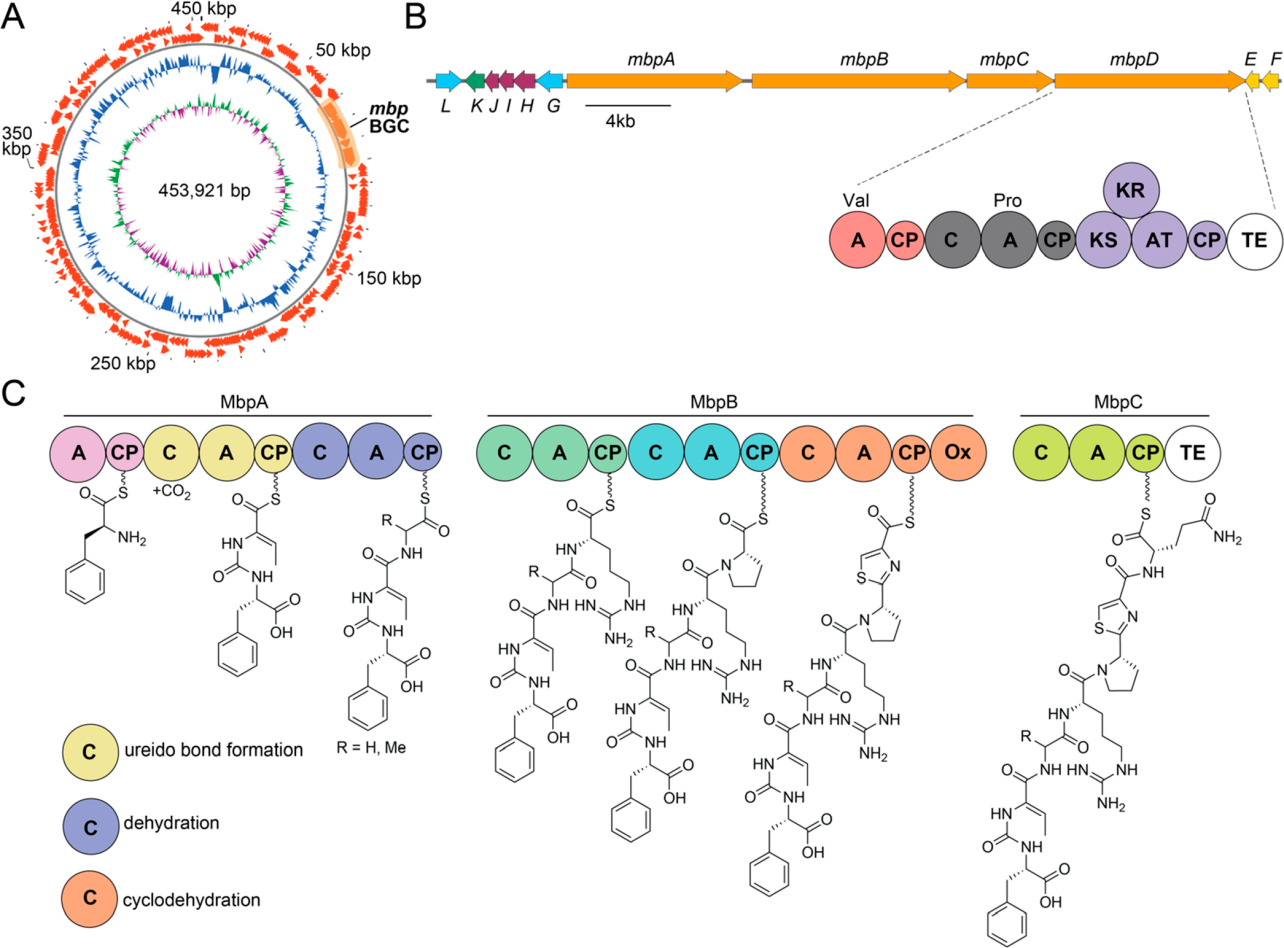
(A) Map of the 454 kbp *Microbulbifer* sp. MKSA007 plasmid bearing the *mbp* BGC. From outside in predicted open reading frames (ORFs) on the leading and lagging DNA strands with the position of the *mbp* BGC highlighted are the normalized plot of GC content (blue) and normalized plot of GC skew (purple/green). (B) *mbp* BGC. (Abbreviations: A, adenylation; CP, carrier protein; C, condensation; Ox, oxidase; TE, thioesterase; KS, ketosynthase; KR, ketoreductase; and AT, acyl transferase). Hybrid NRPS-PKS MbpD is rationalized to contain two NRPS modules with predicted A-domain specificities for Val and Pro and a single PKS module. (C) Inferred assembly line biosynthesis of pseudobulbiferamides mediated by NRPSs MbpA–C. Three C domains, color-coded, conceivably catalyze ureido bond formation, dehydration of a Thr side chain, and Cys to thiazoline oxidation.

Mining the draft genome of the *Microbulbifer* sp. MKSA007 bacterium led to the identification of a second BGC encoding six NRPS modules. Unlike the *mbp* BGC, this second NRPS BGC was present on the bacterial chromosome and not on a plasmid. The organization of this BGC was highly similar to the bulbiferamide-producing *bulb* BGCs that we had previously described from two other marine *Microbulbifer* strains that were likewise cultured from marine sponge microbiomes, *Microbulbifer* sp. MLAF003 and *Microbulbifer* sp. VAAF005 (Figure 3A).^13^ Both, *Microbulbifer* sp. MLAF003 and *Microbulbifer* sp. VAAF005 constitutively produce bulbiferamides in liquid media, while neither strain produces pseudobulbiferamides. Despite the detection of the *bulb* BGC, no bulbiferamides were detected in liquid culture extracts of *Microbulbifer* sp. MKSA007 from which the pseudobulbiferamides were isolated.

**Figure 3:**
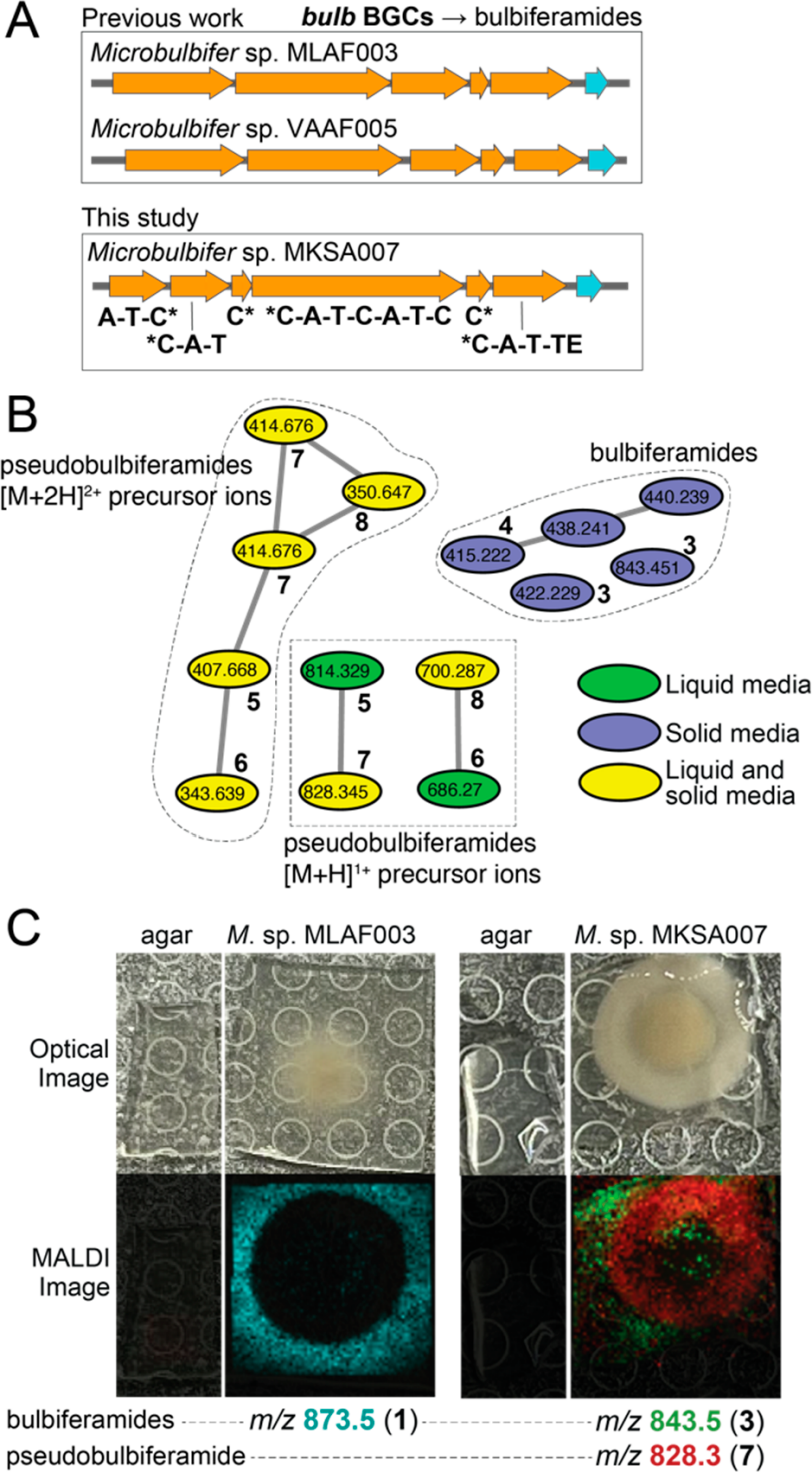
(A) *bulb* BGCs detected in *Microbulbifer* strains, *Microbulbifer* sp. MLAF003, *Microbulbifer* sp. VAAF005,^13^ and *Microbulbifer* sp. MKSA007. The domain organization of the *Microbulbifer* sp. MKSA007 *bulb* NRPSs is denoted. The gene colored cyan encodes a putative transporter. C domains marked by * are split between two polypeptides. (B) Molecular network with nodes denoting bulbiferamides and pseudobulbiferamides detected in *Microbulbifer* sp. MKSA007 culture extracts. Nodes colored green, blue, and yellow are detected in liquid, solid, and both liquid and solid media culture extracts, respectively. The precursor ion *m*/*z* are labeled for each node. Nodes corresponding to different ionization states for both classes of molecules do not cluster. (C) Imaging mass spectrometry denoting differential localization of bulbiferamides and pseudobulbiferamides for bulbiferamide-only producing *Microbulbifer* sp. MLAF003 (left) and bulbiferamide and pseudobulbiferamides producing *Microbulbifer* sp. MKSA007 (right). Different bulbiferamide congeners detected in the two bacterial strains.

To test whether changes in culture conditions might induce the production of *bulb* BGC-encoded bulbiferamides, using molecular networking,^24^ we queried the metabolomes of *Microbulbifer* sp. MKSA007 bacterium when cultured in liquid and in solid media. In both culture conditions, we detected the constitutive production of pseudobulbiferamides. In line with the presence of the *bulb* BGC in the chromosomal DNA, the production of bulbiferamides was indeed detected but only when *Microbulbifer* sp. MKSA007 was cultured on solid media; bulbiferamide production was not detected in liquid media for this strain (Figure 3B). These data establish that *Microbulbifer* sp. MKSA007 can produce two distinct families of ureidopeptide natural products: the hexapeptidic bulbiferamides that are derived from the chromosome encoded *bulb* BGC and the heptapeptidic pseudobulbiferamides that are derived from the plasmid encoded *mbp* BGC. Both families of molecules are produced in solid media, while only the pseudobulbiferamides are produced in liquid media.

The functional relevance, if any, of the coproduction of bulbiferamides and pseudobulbiferamides by the same bacterial strain, and how these two families of ureidopeptidic natural products influence each other’s production is presently not clear. Using imaging mass spectrometry, we detect that bulbiferamides produced by both bulbiferamide-only producing *Microbulbifer* sp. MLAF003 and bulbiferamide and pseudobulbiferamides producing *Microbulbifer* sp. MKSA007 are excreted out of the bacterial colony into the surrounding media. In contrast, pseudobulbiferamides are largely retained within the bacterial colony (Figure 3C). Hence, the two families of NRPS-derived ureidopeptidic natural products are fated to occupy different physical regions in *Microbulbifer* colonies. We know that pseudovibriamides mediate swarming behavior in marine *Pseudovibrio* bacteria;^8^ it is thus conceivable that bulbiferamides and/or pseudobulbiferamides also play fundamentally important roles in *Microbulbifer* organismal biology. The yet to be developed tools for the genetic manipulation of *Microbulbifer* bacteria will be instrumental in answering this lingering question and in the assignment of the physiological role(s) for these two classes of molecules in the *Microbulbifer* genus. With no detectable activity for either class of molecules against Gram-positive or Gram-negative bacteria, it is unlikely that these molecules are mediating competitive interactions in marine sponge microbiomes.

## Experimental Section

### General Experimental Procedures

Optical rotation, circular dichroism, and UV spectra were measured on a JASCO J-815 spectropolarimeter (JASCO). One-dimensional (1D) and two-dimensional (2D) NMR spectra were recorded on a Bruker Avance IIIHD 700 MHz NMR. The ^1^H and ^13^C NMR chemical shifts were referenced to the solvent peaks for DMSO-d6 at δ_H_ 2.50 and δ_C_ 39.52. High-resolution electrospray ionization mass data were recorded using a 1290 Infinity II ultraperformance liquid chromatography (UPLC, Agilent Technologies) coupled to a ImpactII ultrahigh-resolution Q-ToF mass spectrometer equipped with an electron spray ionization (ESI) source (Bruker Daltonics). A Kinetex C18 reversed phase UPLC column (50 × 2.1 mm, 1.7 μm) was used for chromatographic separation. Data were acquired in the positive ionization mode with *m*/*z* 50–2000 Da. Low-resolution electrospray ionization mass data were recorded on a 1260 Infinity high performance liquid chromatography (HPLC, Agilent Technologies) coupled to a amaZon mass spectrometer equipped with an electron spray ionization (ESI) source (Bruker Daltonics). A Poroshell 120 EC-C18 reverse phase HPLC column (100 × 4.6 mm, 5.0 μm) was used for chromatographic separation. Data were acquired in negative ionization mode with *m*/*z* 50–2000 Da. Analytical thin-layer chromatography (TLC) was carried out on silica gel 60 F254 aluminum-backed TLC plates (Merck), with compounds visualized using short-wave UV (254 nm) and by spraying with phosphomolybdic acid reagent followed by heating. Open column chromatography (CC) was performed over silica gel (60–200 mesh, SiliaFlash) and ODS (50 μm, Merck). Semipreparative HPLC was performed on an Agilent 1260 Infinity II HPLC system equipped with a VWD detector, using a Luna C18 reverse phase column (250 × 10 mm, 5 μm). All solvents used in CC and HPLC were analytical grade (VWR) and HPLC grade (Fisher and Sigma-Aldrich), respectively.

### Liquid Fermentation, Extraction, and Compound Purification

The isolation of *Microbulbifer* sp. MKSA007 used in this study from a marine sponge has been previously described.^11^ Liquid fermentation and extraction were the same as previously described.^13^ The extract (3.1 g) was subjected to a silica gel column chromatography using stepwise gradient elution with CH_2_Cl_2_/MeOH (v/v, 20:1, 10:1, 5:1, 1:1, 0:1, each 600 mL) to yield 10 fractions (1–10). Fractions 7–10 (210 mg) were combined and further fractionated using semipreparative HPLC [C_18_ column, 250 × 10 mm, 5 μm, 10–100% MeCN/H_2_O (+0.1% v/v TFA) gradient across 25 min at 2 mL/min flow rate] to yield **5** (7.3 mg), **6** (0.9 mg), **7** (1.2 mg), and **9** (2.5 mg).

#### Pseudobulbiferamide A (**5**)

Yellow oil; [α]^25^_D_= −159.5 (*c* 0.2, MeOH); UV (MeOH) λ_max_ (log ε) 200 (4.91, end absorption), 209 (4.80); ECD (MeOH) λ_max_ (Δε) 219 (3.43), 235 (−3.04); ^1^H and ^13^C NMR data, Table 1; HRESIMS *m*/*z* 814.3294 [M + H]^+^ (calcd for C_35_H_48_N_11_O_10_S, 814.3301).

#### Pseudobulbiferamide B (**6**)

Yellow oil; [α]^25^_D_= −96.9 (*c* 0.1, MeOH); UV (MeOH); λ_max_ (log ε) 200 (4.84, end absorption), 207 (4.76); ECD (MeOH) λ_max_ (Δε) 220 (7.92), 241 (−5.83); ^1^H and ^13^C NMR data, Table S1; HRESIMS *m*/*z* 686.2714 [M + H]^+^ (calcd for C_30_H_40_N_9_O_8_S, 686.2715).

#### Pseudobulbiferamide C (**7**)

Yellow oil; [α]^25^_D_= −181.1 (*c* 0.2, MeOH); UV (MeOH) λ_max_ (log ε) 200 (4.66, end absorption), 205 (4.60); ECD (MeOH) λ_max_ (Δε) 210 (−12.69), 230 (−11.96); ^1^H and ^13^C NMR data, Table 1; HRESIMS *m*/*z* 828.3445 [M + H]^+^ (calcd for C_36_H_50_N_11_O_10_S, 828.3457).

#### Pseudobulbiferamide D (**8**)

Positive HRESIMS *m*/*z* 700.2872 [M + H]^+^ (calcd for C_31_H_42_N_9_O_8_S, 700.2872).

#### Compound **9**

Yellow oil; [α]^25^_D_= −48.3 (*c* 0.1, MeOH); UV (MeOH) λ_max_ (log ε) 200 (4.42, end absorption), 207 (4.33); ECD (MeOH) λ_max_ (Δε) 203 (16.09), 220 (10.11); ^1^H and ^13^C NMR data, Table S1; HRESIMS *m*/*z* 293.1122 [M + H]^+^ (calcd for C_14_H_17_N_2_O_5_, 293.1132).

### Solid Cultivation and Extraction

*Microbulbifer* sp. MKSA007 was routinely cultured on three MB1/2 agar plates each containing 20 mL MB1/2 with 0.5% agar for 5 days at 30 °C. The agar media and cells were combined and frozen at −80 °C, followed by lyophilization. The dried media and cells were extracted with 40 mL MeOH and acetone by sonication for 15 min, separately. The MeOH and acetone extracts were recovered by filtration, combined, and dried in vacuo to yield an organic extract. The MB1/2 agar blank extract was prepared the same way.

### Marfey’s Analysis

Compounds **5**, **7**, and **9**, as well as amino acid standards were derivatized by Marfey’s reaction as previously described.^13^

### MALDI-ToF Imaging Mass Spectrometry

A 1 μL inoculum of each isolate *Microbulbifer* sp. MLAF003 and *Microbulbifer* sp. MKSA007 (OD_600_ of 0.05 and 0.01) was spotted on MB1/2 agar (10 mL agar poured in a standard 10 cm Petri dish) and incubated at 30 °C for 4 and 3 days, respectively. The agar containing the colony and the uninoculated section of the agar were then excised from the Petri dish and gently placed on a Bruker Daltonics ground steel MALDI 96 anchor target plate. The recrystallized and finely powdered matrix of 1:1 2,5-dihydroxybenzoic acid:α-cyano-4-hydroxycinnaimic acid was applied to the sample using the sieve method and dried for ∼8 h at 30 °C and ∼1 h in a desiccator. For calibration of the instrument, peptide calibration standard (Bruker Daltonics Inc., 8206195) was mixed with matrix and spotted on the target plate next to the dried agar slice. All samples were imaged using rapifleX MALDI-TOF at a scan range of 25 × 25 μm resulting in a field size of 104 × 104 μm in reflectron positive mode in the mass range from 200 to 3520 *m*/*z*. The laser intensity and detector gain were optimized for individual experiments, and spectra were acquired using a M5 defocus as laser setting. Data were analyzed using Bruker Daltonics FlexImaging 5.0.

## Data Availability

The *Microbulbifer* sp. MKSA007 genome has been deposited in GenBank with the bioproject accession number PRJNA941849. Mass spectrometry fragmentation spectra for pseudobulbiferamides have been deposited to spectral databases as outlined in Table S2. NMR spectra for molecules **5**–**7** and **9** have been deposited to the NP-MRD database (NP0331779, NP0331780, NP0331778, and NP0331781, respectively).

## Supporting Information Available

High-resolution mass spectra for **5**–**9**; ^1^H, ^13^C, DEPT, HSQC, HMBC, COSY, TOCSY, and ROESY NMR spectra for **5**–**7** and **9**; and tables of NMR data for **6** and **9** (PDF)
