# Supplementary material for: Pseudobulbiferamides: Plasmid-Encoded Ureidopeptide Natural Products with Biosynthetic Gene Clusters Shared Among Marine Bacteria of Different Genera
Source: J Nat Prod. 2023 Sep 15;86(10):2414–20. doi: 10.1021/acs.jnatprod.3c00595 (PMC10616845; doi:10.1021/acs.jnatprod.3c00595)
Supplement: Supplementary file 1 — np3c00595_si_001.pdf [file np3c00595_si_001.pdf]

SUPPLEMENTARY INFORMATION FOR:

Pseudobulbiferamides: plasmid-encoded ureidopeptide natural products with biosynthetic gene clusters shared among marine bacteria of different genera

Weimao Zhong,[1] Nicole M. Aiosa,[1,†] Jessica M. Deutsch,[1,†] Neha Garg,[1,2] and Vinayak Agarwal[1,3,*]

[1]School of Chemistry and Biochemistry, Georgia Institute of Technology, Atlanta, GA 30332, USA

[2]Center for Microbial Dynamics and Infection, Georgia Institute of Technology, Atlanta, GA, 30332, USA

[3]School of Biological Sciences, Georgia Institute of Technology, Atlanta, GA 30332, USA

[†]Equal contribution authors

*Correspondence: vagarwal@gatech.edu; Ph: (+1)404-385-3798

TABLE OF CONTENTS

Table S1–S2

Figures S1–S45

Supplementary references

SUPPLEMENTARY TABLES

Table S1. ^{13}C (176 MHz) and ^{1}H (700 MHz) NMR data of compounds **6** and **9** in DMSO-d_6 (J in Hz, δ in ppm).

		**6**		**9**	
Residue	No.	δ_C, type[a]	δ_H (J, Hz)[b]	δ_C, type[a]	δ_H (J, Hz)[b]
Phe[1]	1	173.8, C		173.5, C	
	2	54.6, CH	4.39, br s	53.8, CH	4.39, ddd (7.8, 7.5, 5.3)
	3	37.7, CH_2	3.04, dd (13.7, 5.3)	37.5, CH_2	3.04, dd (13.7, 5.3)
			2.89, ddd (13.7, 7.5)		2.89, dd (13.7, 7.5)
	4	137.8, C		137.2, C	
	5/9	129.4, CH	7.16, d (7.3)	129.3, CH	7.20, d (7.6)
	6/8	128.1, CH	7.27, t (7.6)	128.2, CH	7.28, t (7.6)
	7	126.4, CH	7.21, t (7.3)	126.5, CH	7.22, t (7.4)
	NH		6.47, br s		6.44, d (8.1)
Ureido	CO	nd		154.6, C	
Dhb[2]	1	165.5, C		166.3, C	
	2	132.4, C		119.5, C	
	3	121.3, CH	5.90, q (7.4)	127.8, CH	6.30, q (7.1)
	4	12.2, CH_3	1.61, d (7.0)	13.7, CH_3	1.58, d (7.1)
	NH		7.90, q		7.58, s
Gly[3]	1	169.2, C			
	2	42.3, CH_2	3.73, overlap		
			3.63, dd (17.4, 6.3)		
	NH		8.08, t (6.1)		
Arg[4]	1	170.5, C			
	2	50.2, CH	4.53, m		
	3	28.1, CH_2	1.74, m		
			1.65, m		
	4	24.7, CH_2	1.55, m		
			1.53, m		
	5	40.5, CH_2	3.08, m		
	6	156.7, C			
	αNH		8.11, d (7.5)		
	δNH		nd		
	εNH		nd		
Pro[5]-	1	173.5, C			
Thz[6]	2	58.3, CH	5.30, dd (8.1, 2.5)		
	3	31.4, CH_2	2.20, m		
			2.11, m		
	4	24.0, CH_2	2.00, m		
			1.93, m		
	5	46.8, CH_2	3.08, overlap		
	1′	168.5, C			
	2′	149.0, C			
	3′	128.3, CH	8.27, s		

[a]Recorded at 176 MHz. [b]Recorded at 700 MHz. [nd]Not detected.

Table S2. Identification numbers for MS/MS spectra deposition for pseudobulbiferamides in the GNPS library

**5**	$[M+H]^{1+}$	CCMSLIB00011427564
**5**	$[M+2H]^{2+}$	CCMSLIB00011427566
**6**	$[M+H]^{1+}$	CCMSLIB00011427563
**6**	$[M+2H]^{2+}$	CCMSLIB00011427567
**7**	$[M+H]^{1+}$	CCMSLIB00011427565
**8**	$[M+H]^{1+}$	CCMSLIB00011427568
**8**	$[M+2H]^{2+}$	CCMSLIB00011427569

Supplementary Figures

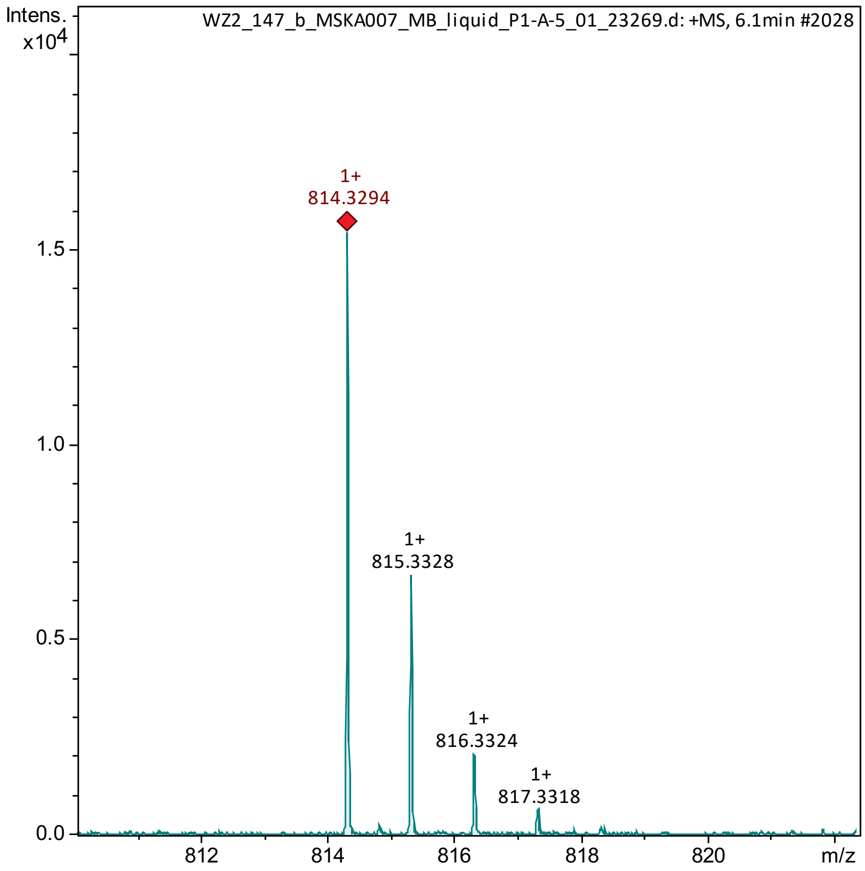

Figure S1. High resolution $[M+H]^{1+}$ MS^1 spectrum for **5**.

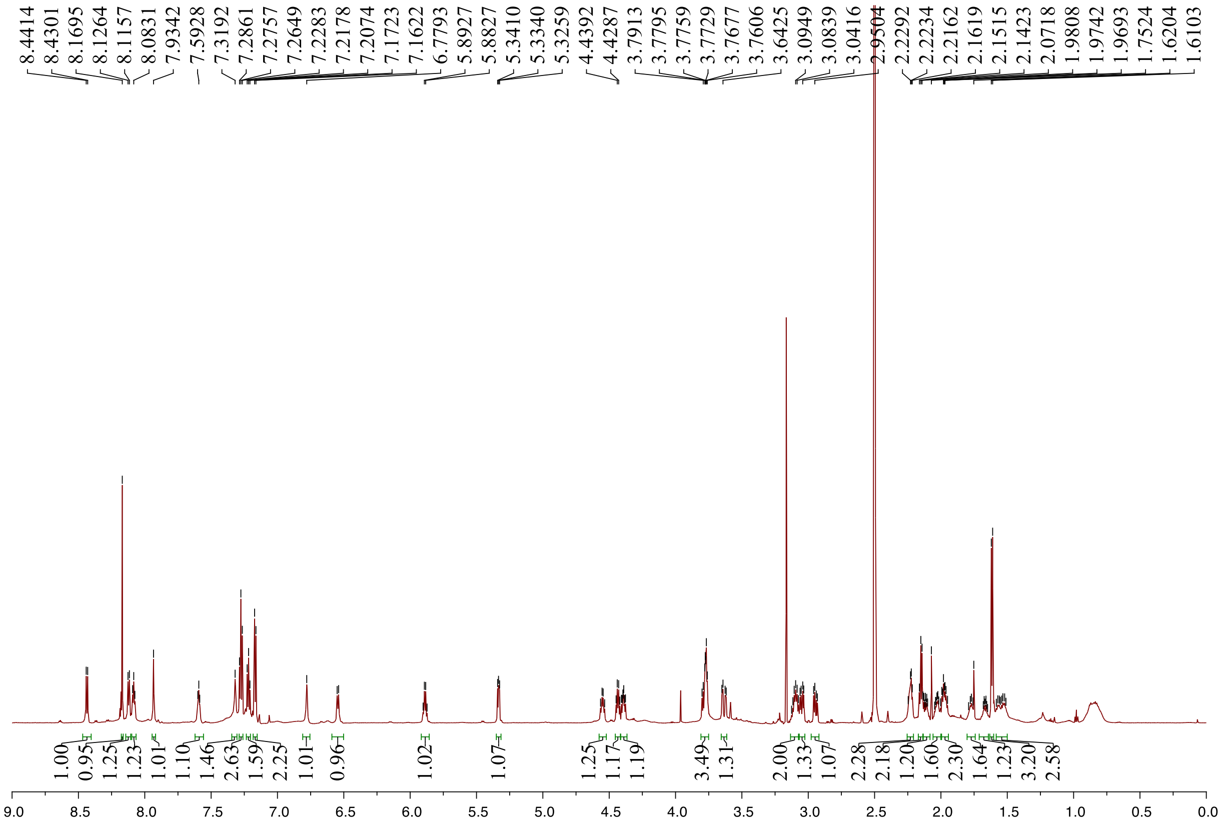

Figure S2. The ^{1}H NMR spectrum of **5** (700 MHz, DMSO-d_6).

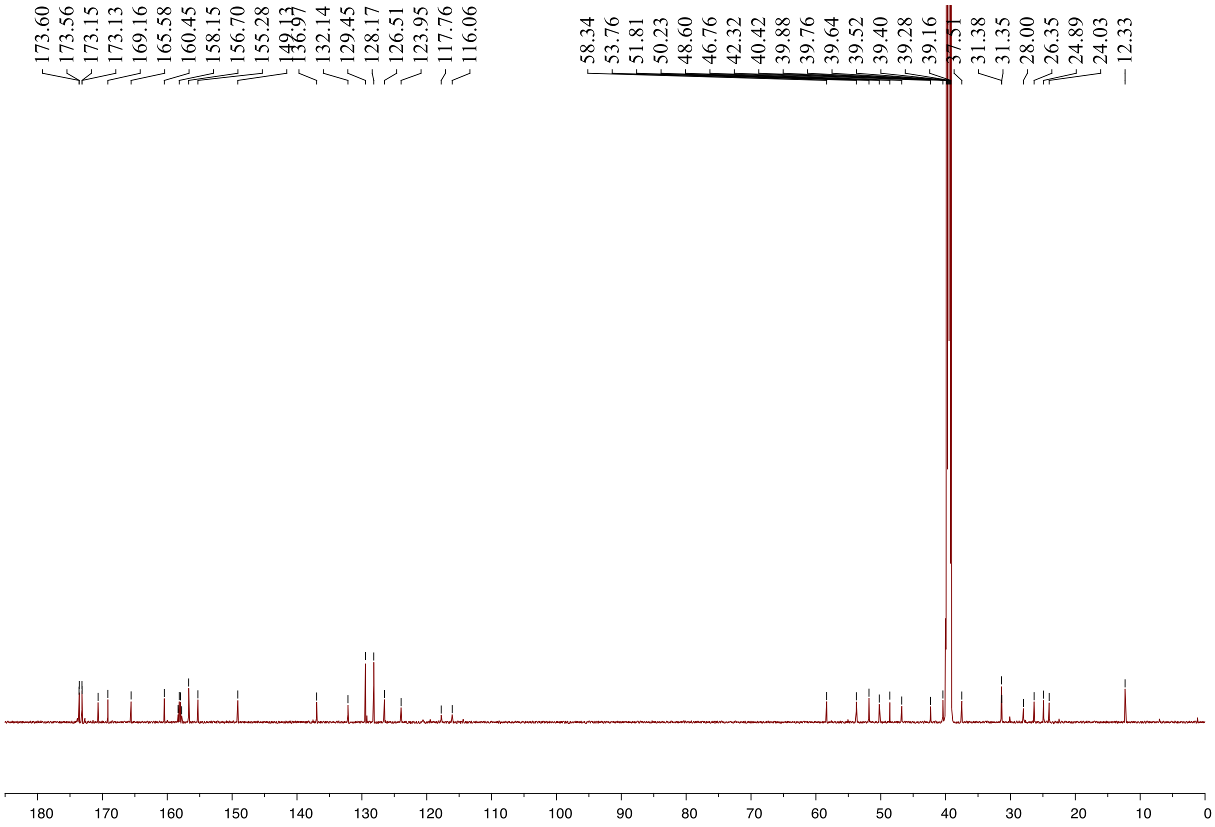

Figure S3. The ^{13}C NMR spectrum of **5** (176 MHz, DMSO-d_6).

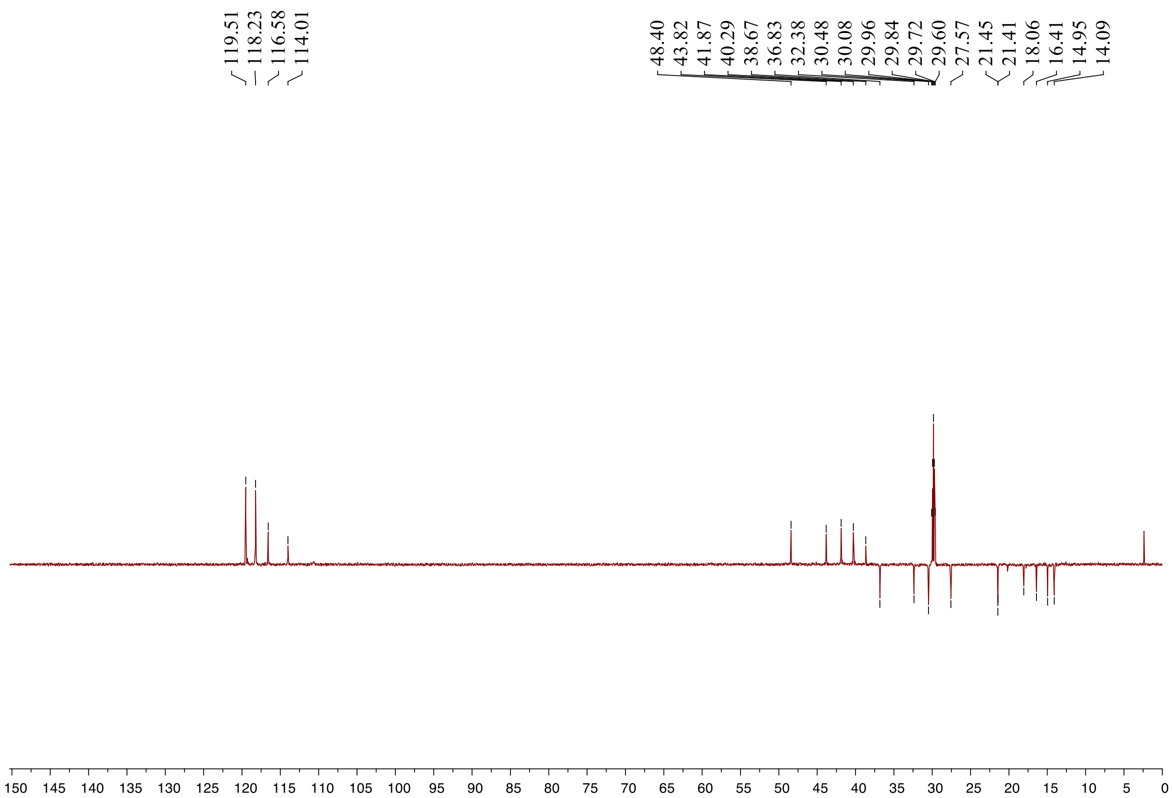

Figure S4. The DEPT135 spectrum of **5** (176 MHz, DMSO-d_6).

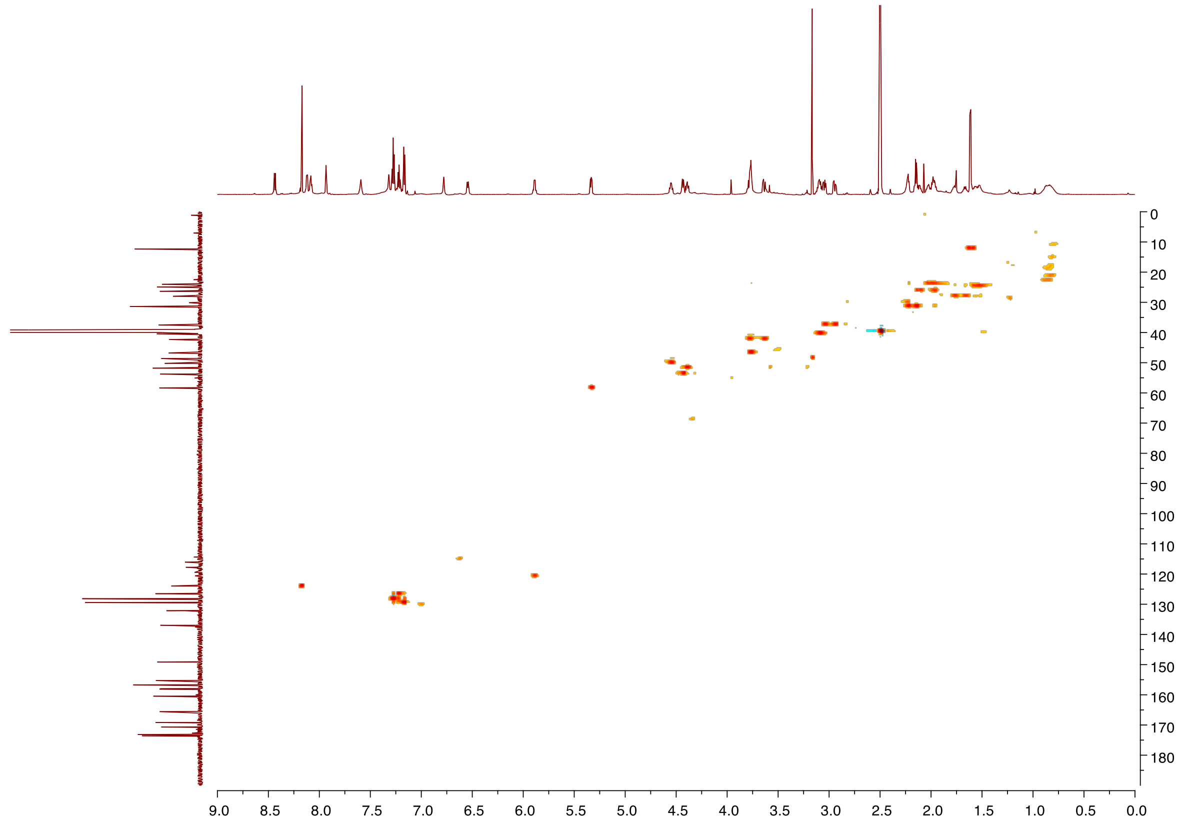

Figure S5. The HSQC spectrum of **5** (700 MHz, DMSO-d_6).

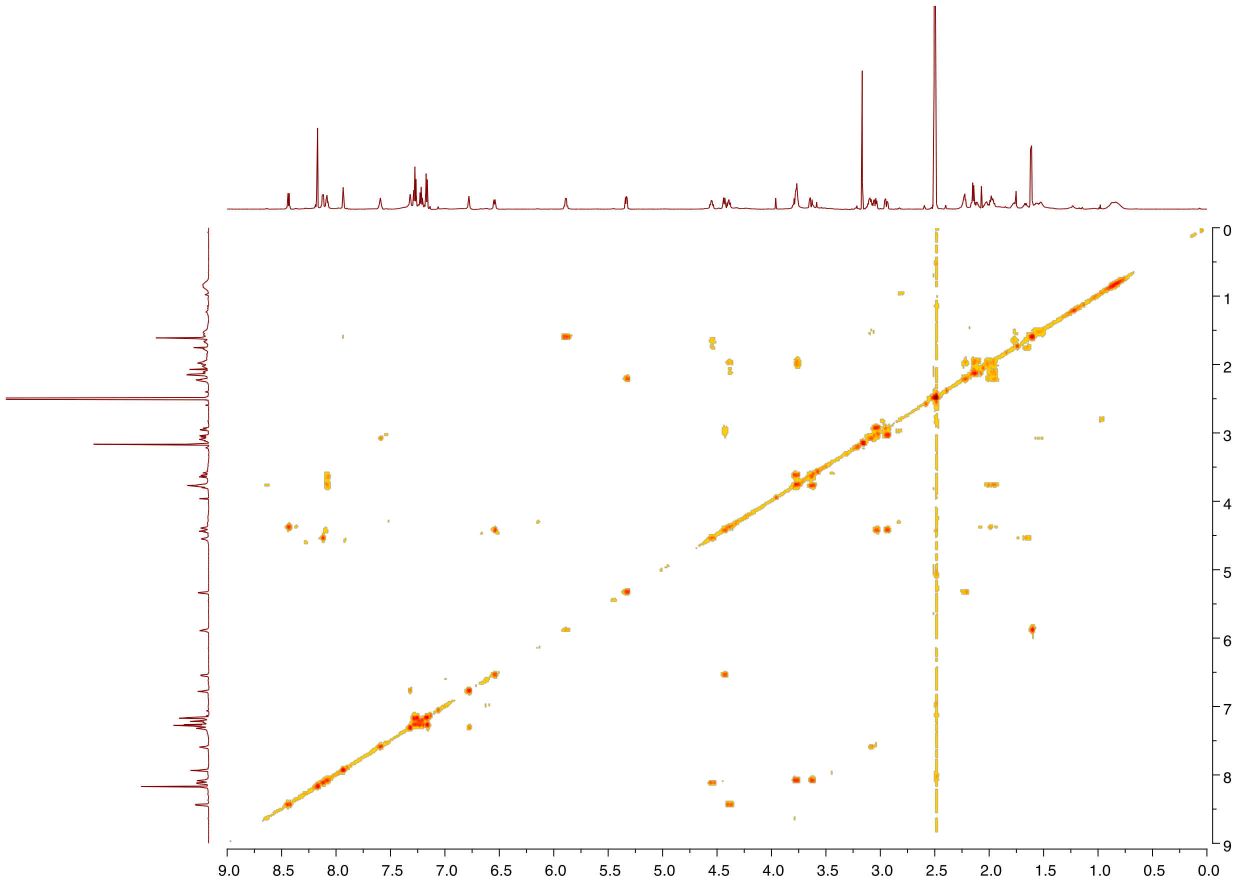

Figure S6. The ^{1}H-^{1}H COSY spectrum of **5** (700 MHz, DMSO-d_6).

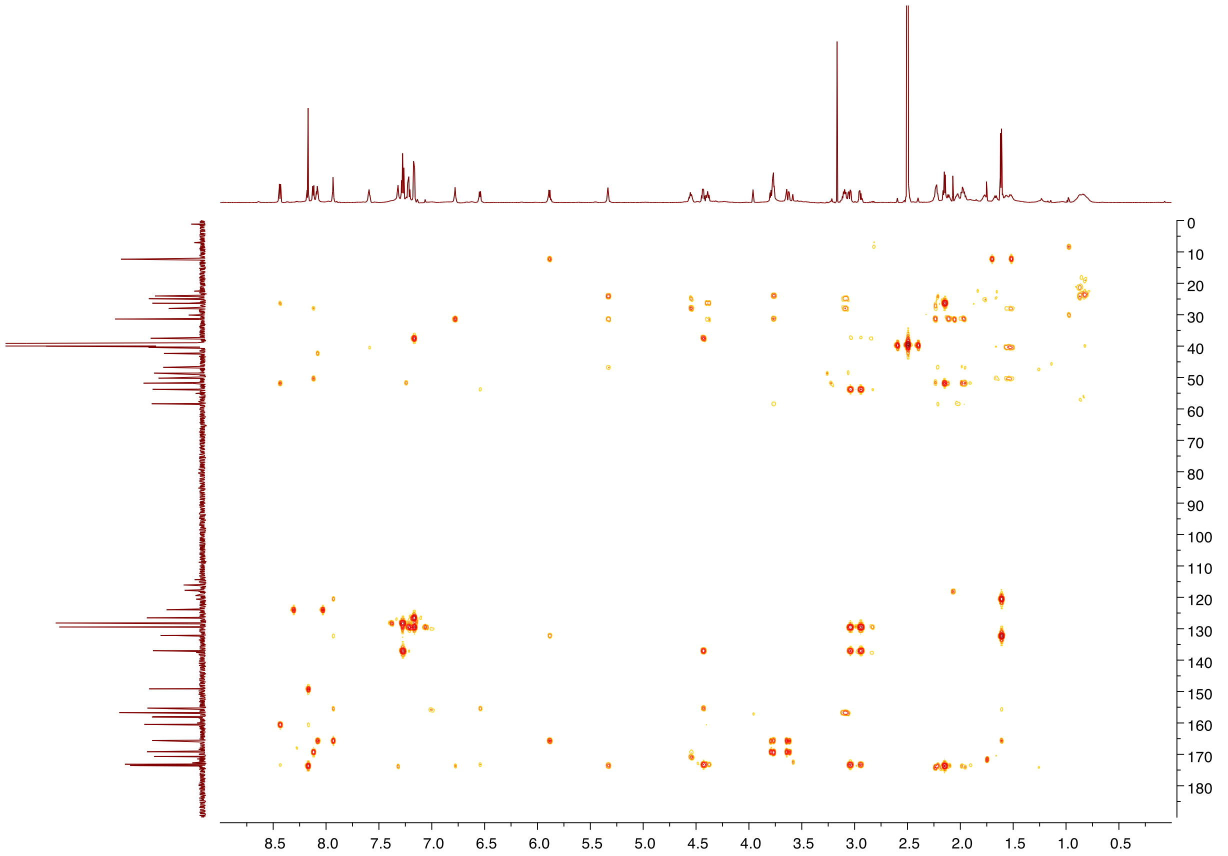

Figure S7. The HMBC spectrum of **5** (700 MHz, DMSO-d_6).

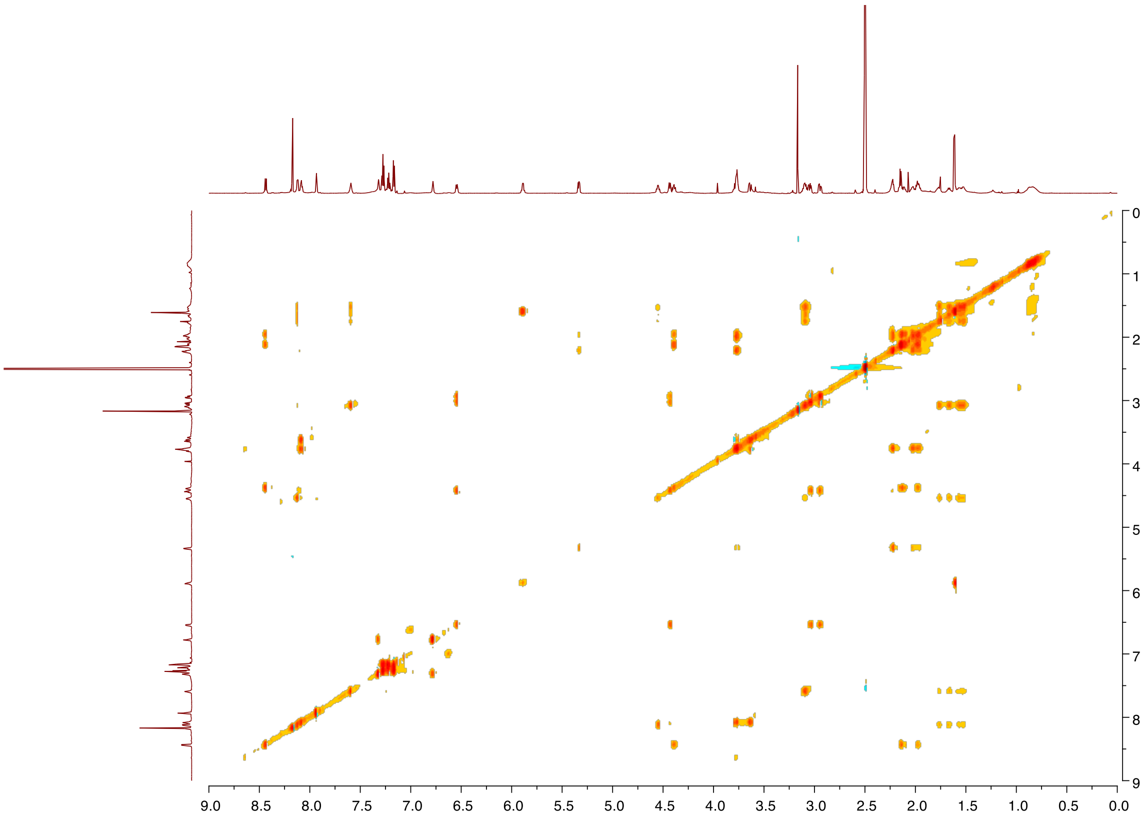

Figure S8. The TOCSY spectrum of **5** (700 MHz, DMSO-d_6).

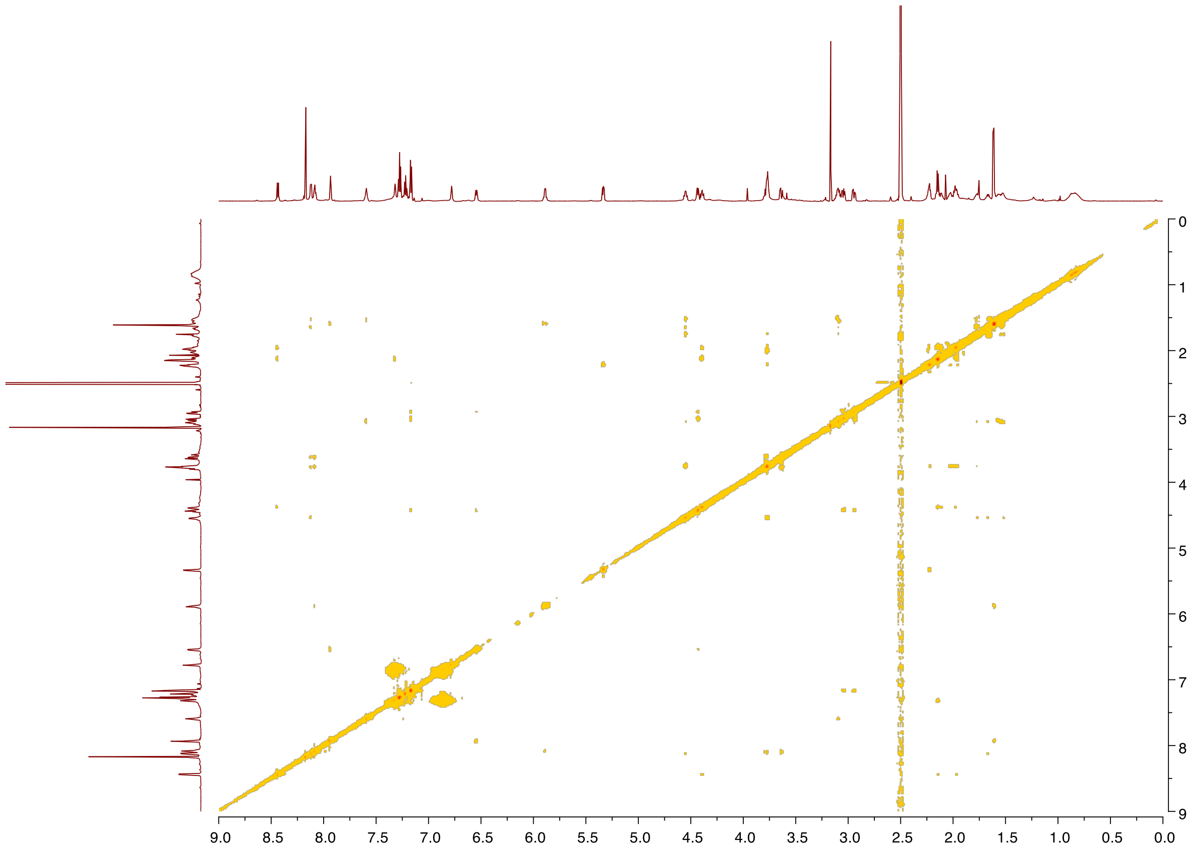

Figure S9. The ROESY spectrum of **5** (700 MHz, DMSO-d_6).

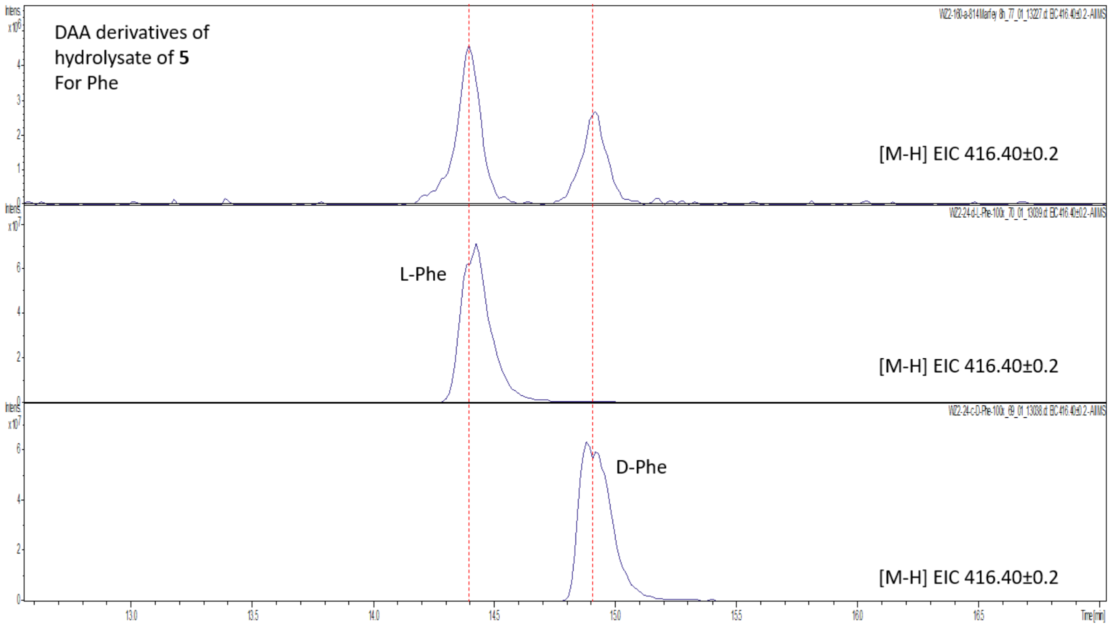

Figure S10. Marfey's analysis to determine the absolute configuration of the Phe residue in **5**. Extracted ion chromatograms (EICs) demonstrating the retention time of the 2-4-dinitrophenyl-5-L-alanine amide -derivitized (DAA-derivatized) Phe residue resulting from the acid hydrolysis of **5** (top), retention time of DAA-derivatized standard of L-Phe (middle), and the retention time of the similarly derivatized standard of D-Phe (bottom). Of note, although only one Phe is present in **5**, both a major peak of L-Phe and a minor one of D-Phe were detected by retention time matching. We deduced that racemization of L-Phe took place during acidic hydrolysis, which is akin to homophymamide A that has been verified by chemical synthesis, due to their exocyclic amino acid residue structures attached to the ureido bond.[1] Separation was achieved using the Agilent Poroshell EC-C18 (100×4.6 mm, 2.7 µm) column. Mass spectrometry data were acquired in the negative ionization mode.

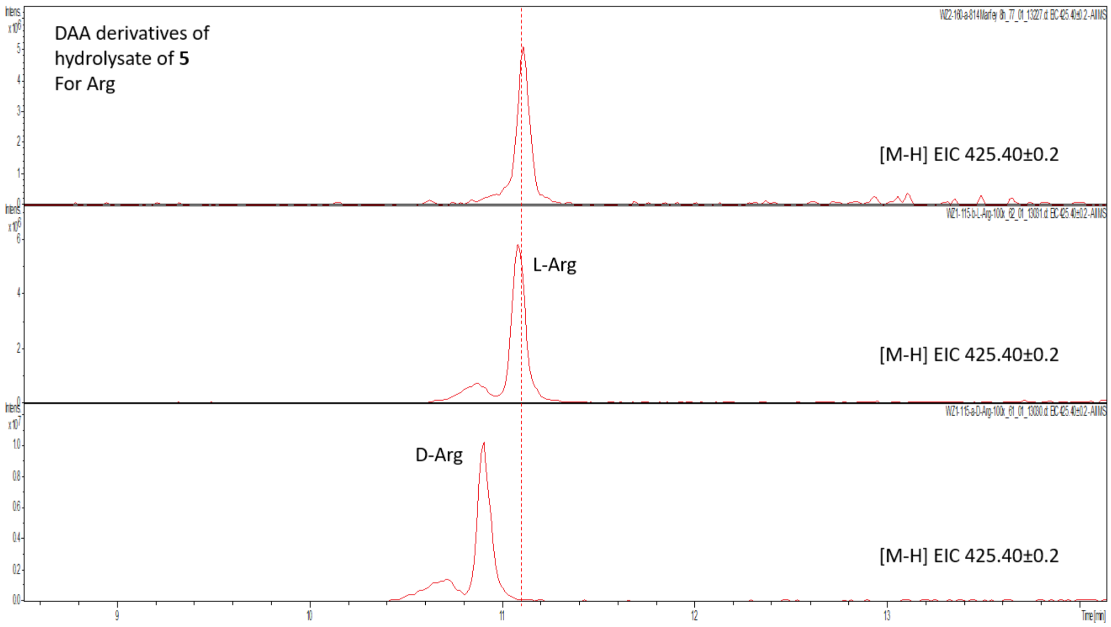

Figure S11. Marfey's analysis to determine the absolute configuration of the Arg residue in **5**. Extracted ion chromatograms (EICs) demonstrating the retention time of the DAA-derivatized Arg residue resulting from the acid hydrolysis of **5** (top), retention time of DAA-derivatized standard of L-Arg (middle), and the retention time of the similarly derivatized standard of D-Arg (bottom). By retention time matching, the Arg residue in **5** was determined to be L-Arg. Separation was achieved using the Agilent Poroshell EC-C18 (100×4.6 mm, 2.7 µm) column. Mass spectrometry data were acquired in the negative ionization mode.

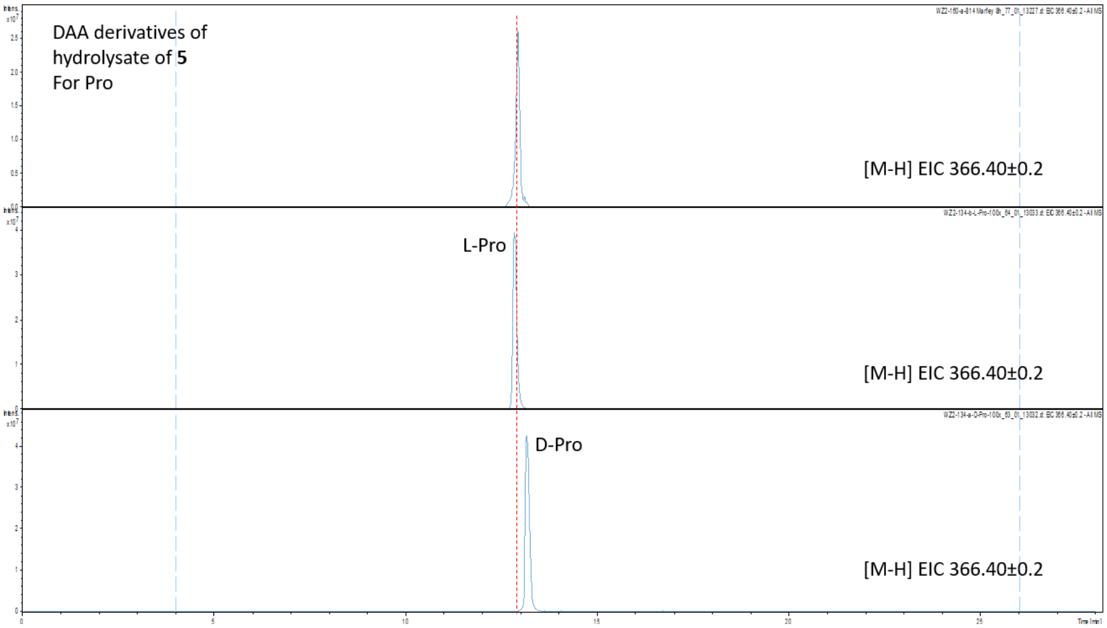

Figure S12. Marfey's analysis to determine the absolute configuration of the Pro residue in **5**. Extracted ion chromatograms (EICs) demonstrating the retention time of the DAA-derivatized Pro residue resulting from the acid hydrolysis of **5** (top), retention time of DAA-derivatized standard of L-Pro (middle), and the retention time of the similarly derivatized standard of D-Pro (bottom). By retention time matching, the Pro residue in **5** was determined to be L-Pro. Separation was achieved using the Agilent Poroshell EC-C18 (100×4.6 mm, 2.7 μm) column. Mass spectrometry data were acquired in the negative ionization mode.

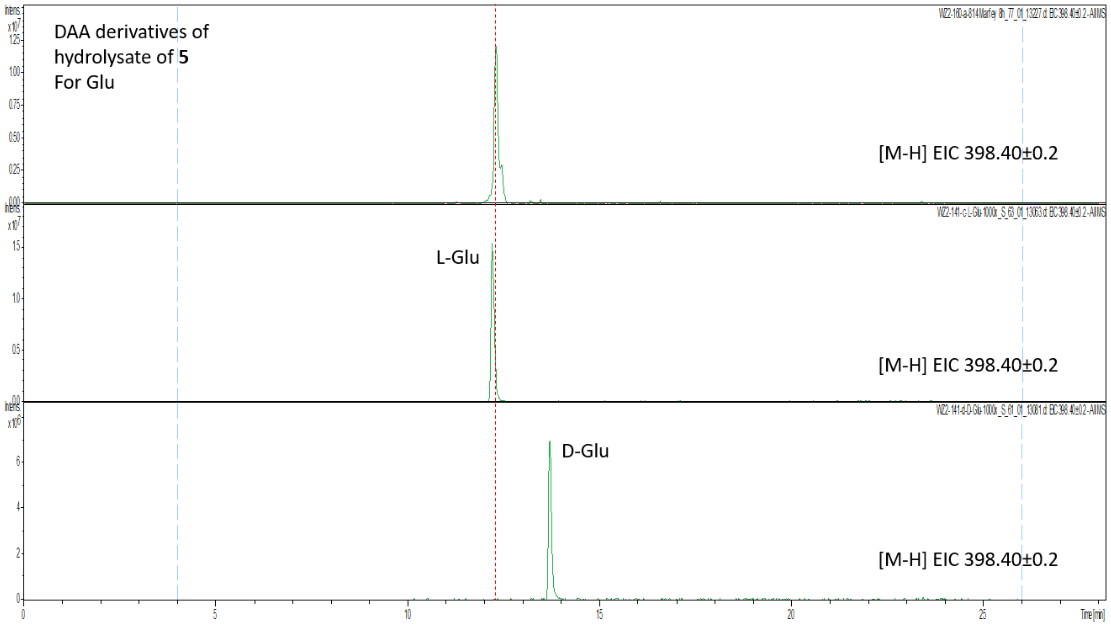

Figure S13. Marfey's analysis to determine the absolute configuration of the Gln residue in **5**. During acid hydrolysis, Gln is converted to Glu. Hence, Glu standards are used here. Extracted ion chromatograms (EICs) demonstrating the retention time of the DAA-derivatized Glu residue resulting from the acid hydrolysis of **5** (top), retention time of DAA-derivatized standard of L-Glu (middle), and the retention time of the similarly derivatized standard of D-Glu (bottom). By retention time matching, the Gln residue in **5** was determined to be L-Gln. Separation was achieved using the Agilent Poroshell EC-C18 (100×4.6 mm, 2.7 μm) column. Mass spectrometry data were acquired in the negative ionization mode.

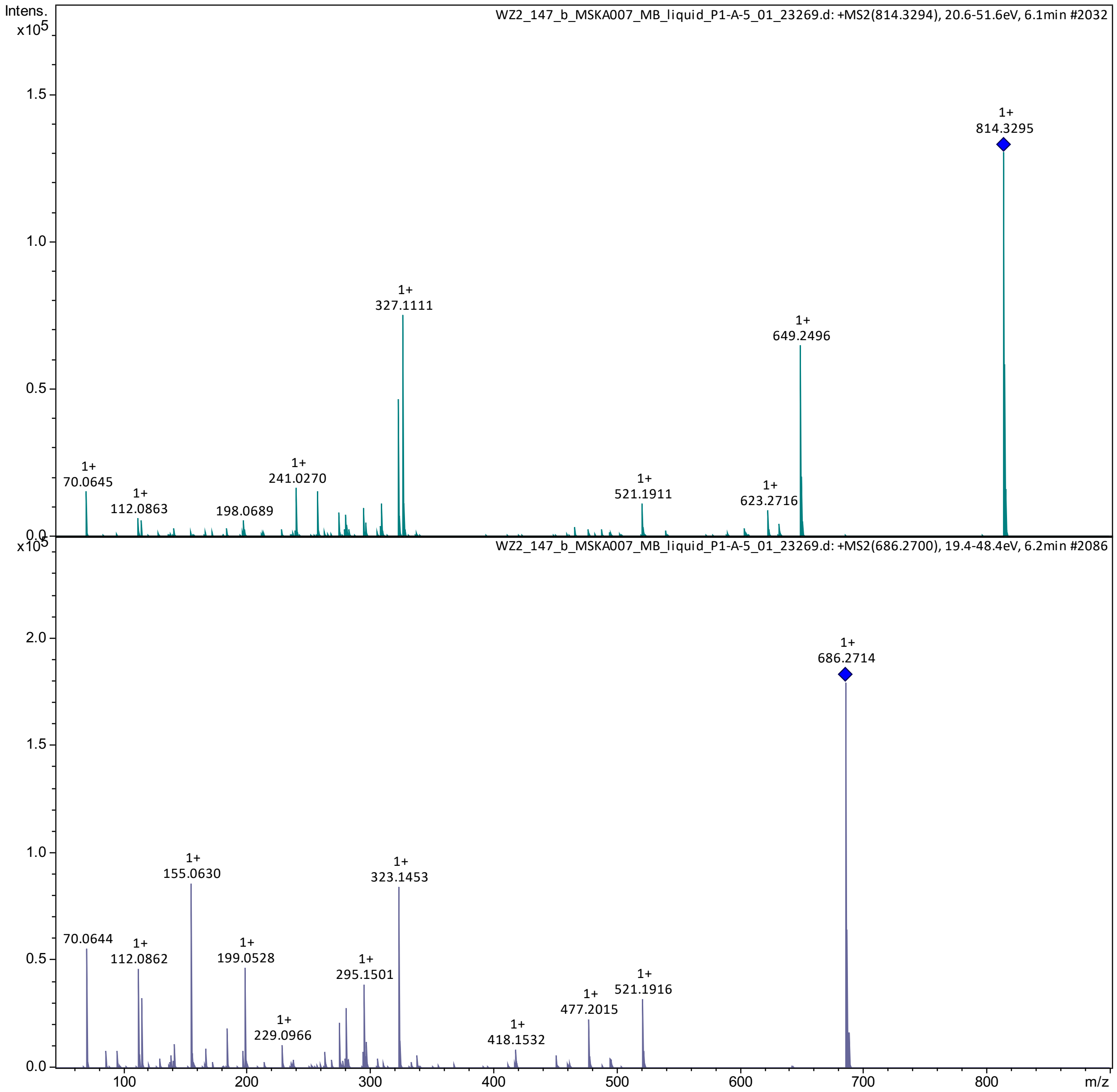

Figure S14. HRMS/MS spectra for **5** (top) and **6** (bottom).

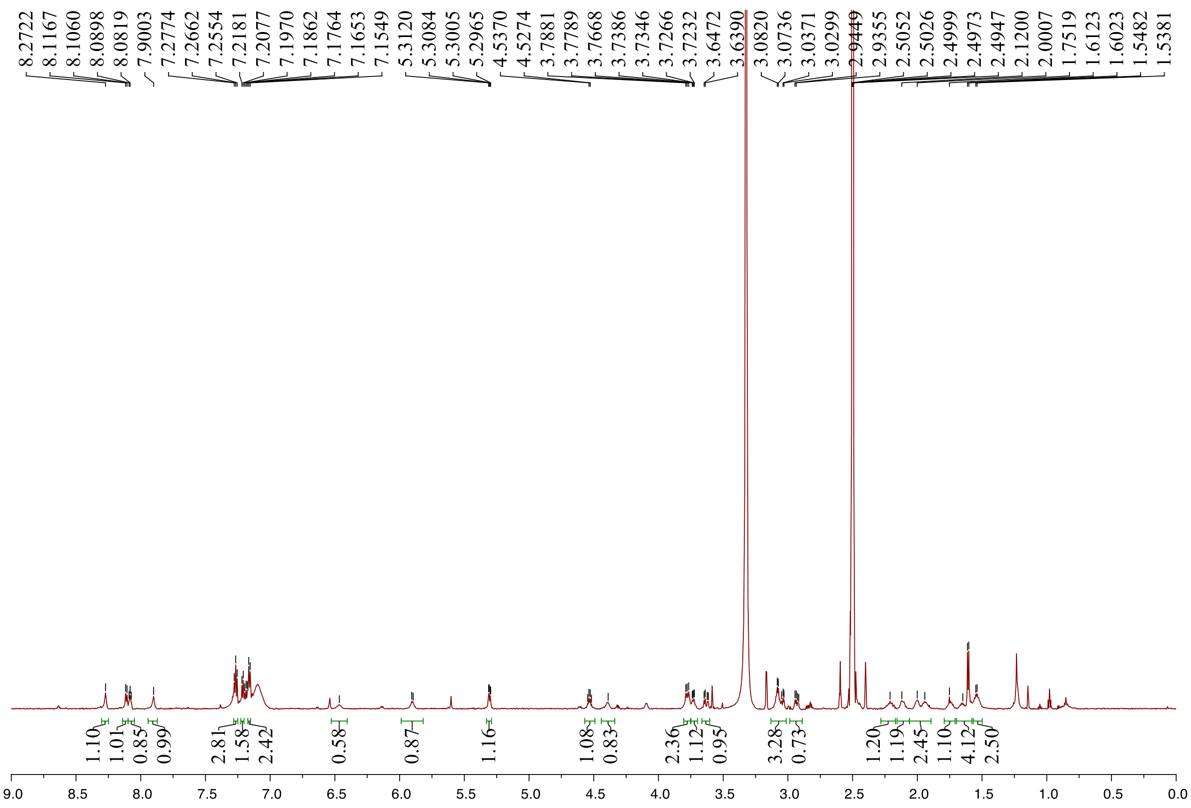

Figure S15. The ^{1}H NMR spectrum of **6** (700 MHz, DMSO-d_6).

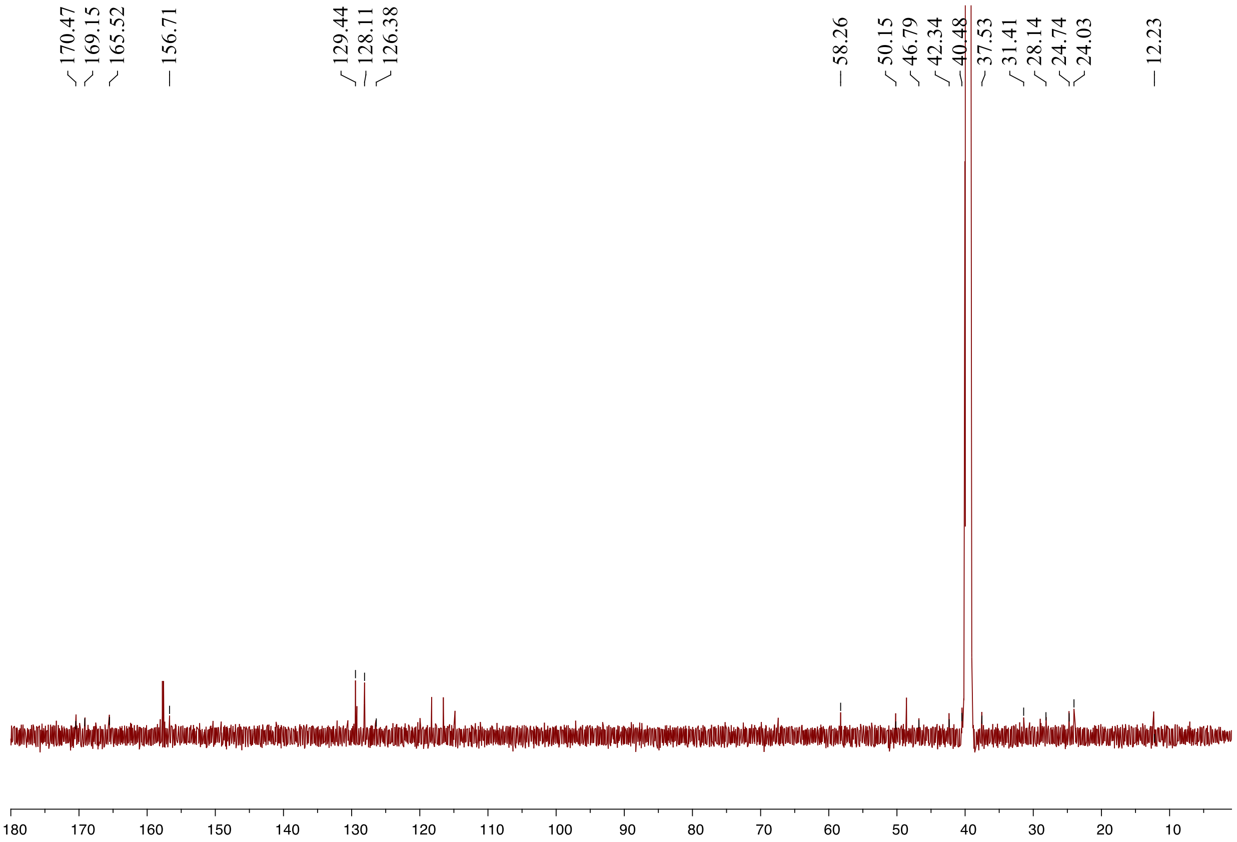

Figure S16. The ^{13}C NMR spectrum of **6** (176 MHz, DMSO-d_6).

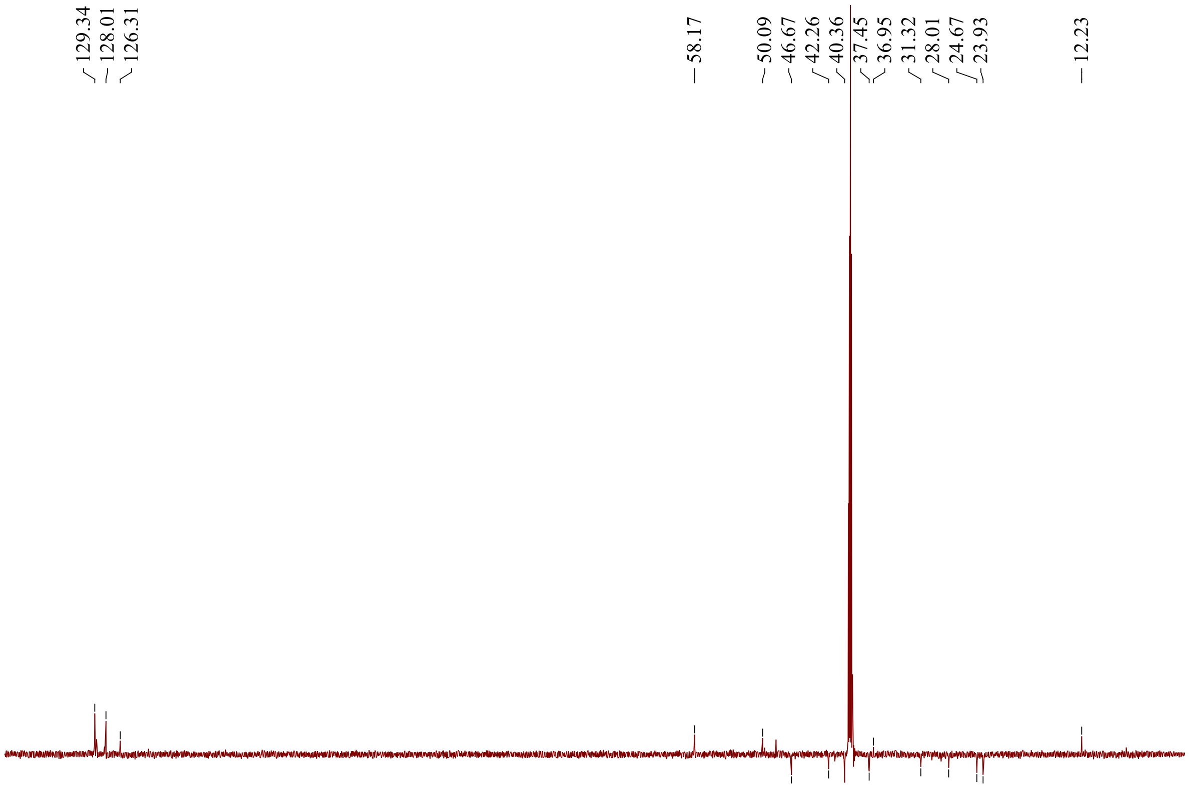

Figure S17. The DEPT135 spectrum of **6** (176 MHz, DMSO-d_6).

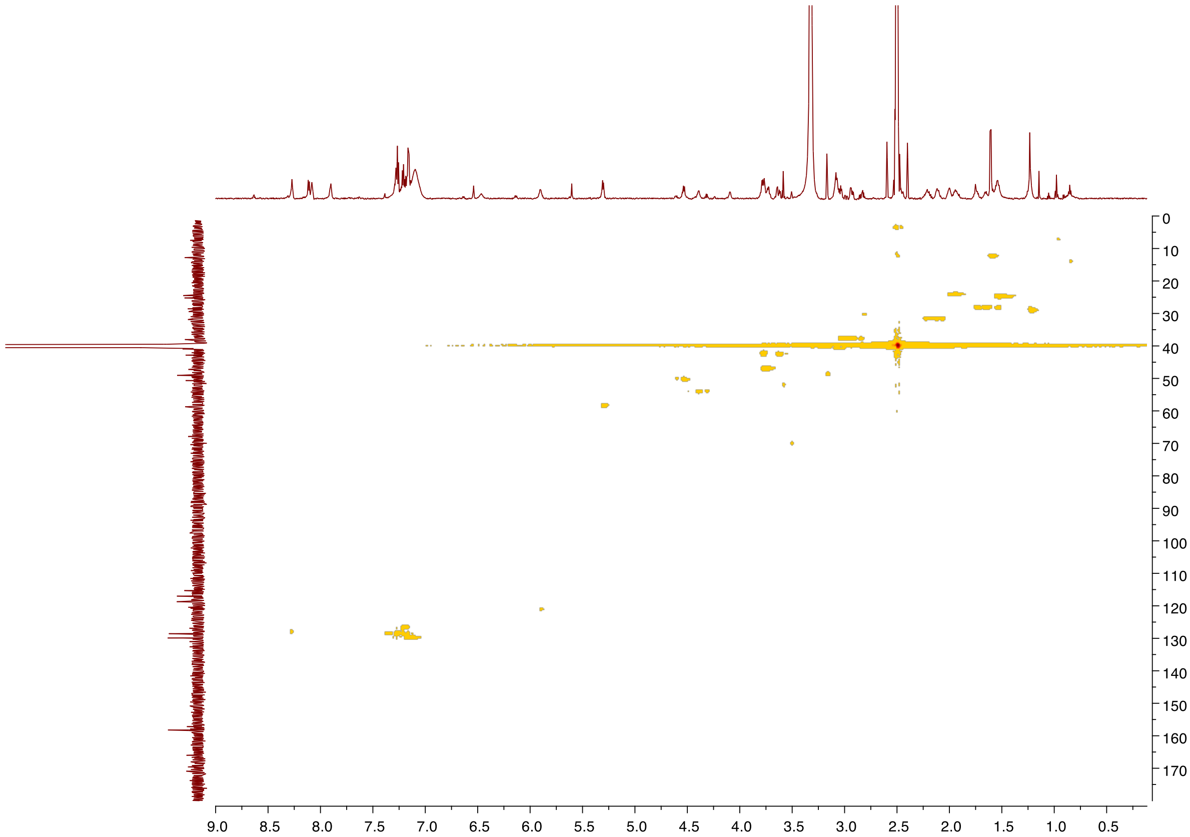

Figure S18. The HSQC spectrum of **6** (700 MHz, DMSO-d_6).

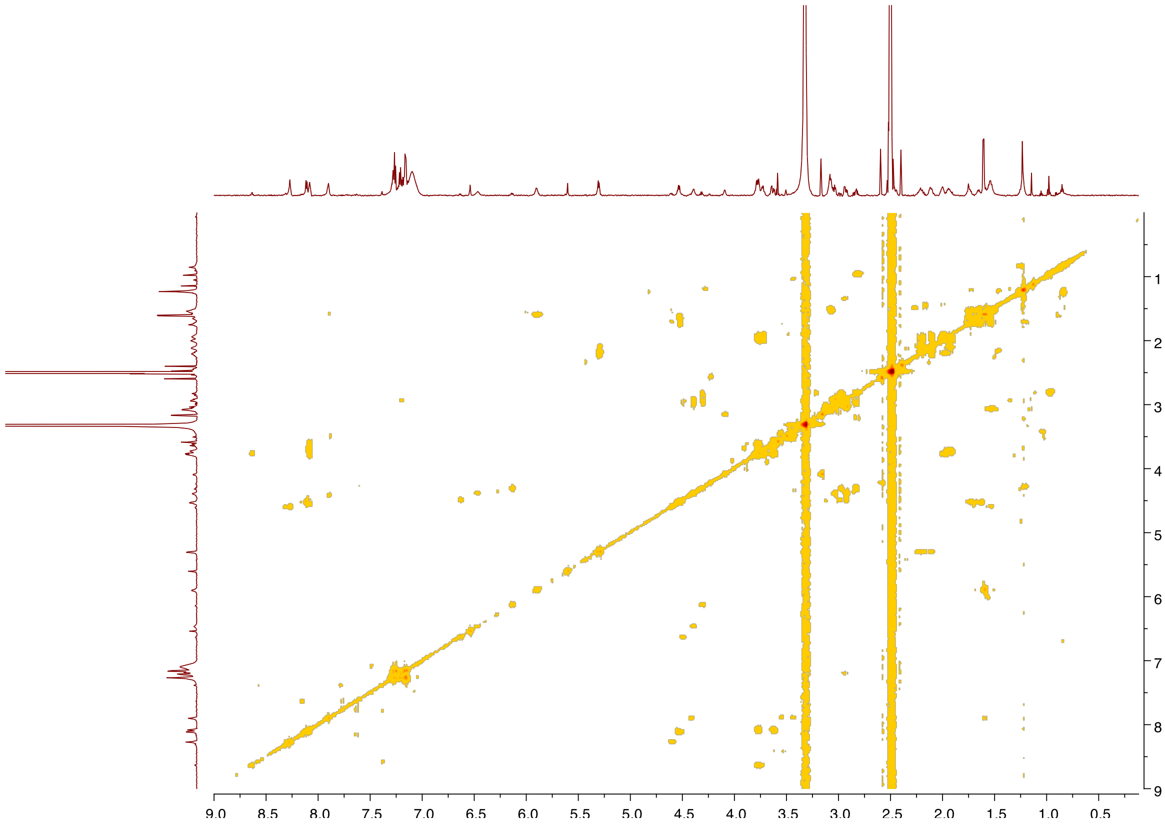

Figure S19. The ^{1}H-^{1}H COSY spectrum of **6** (700 MHz, DMSO-d_6).

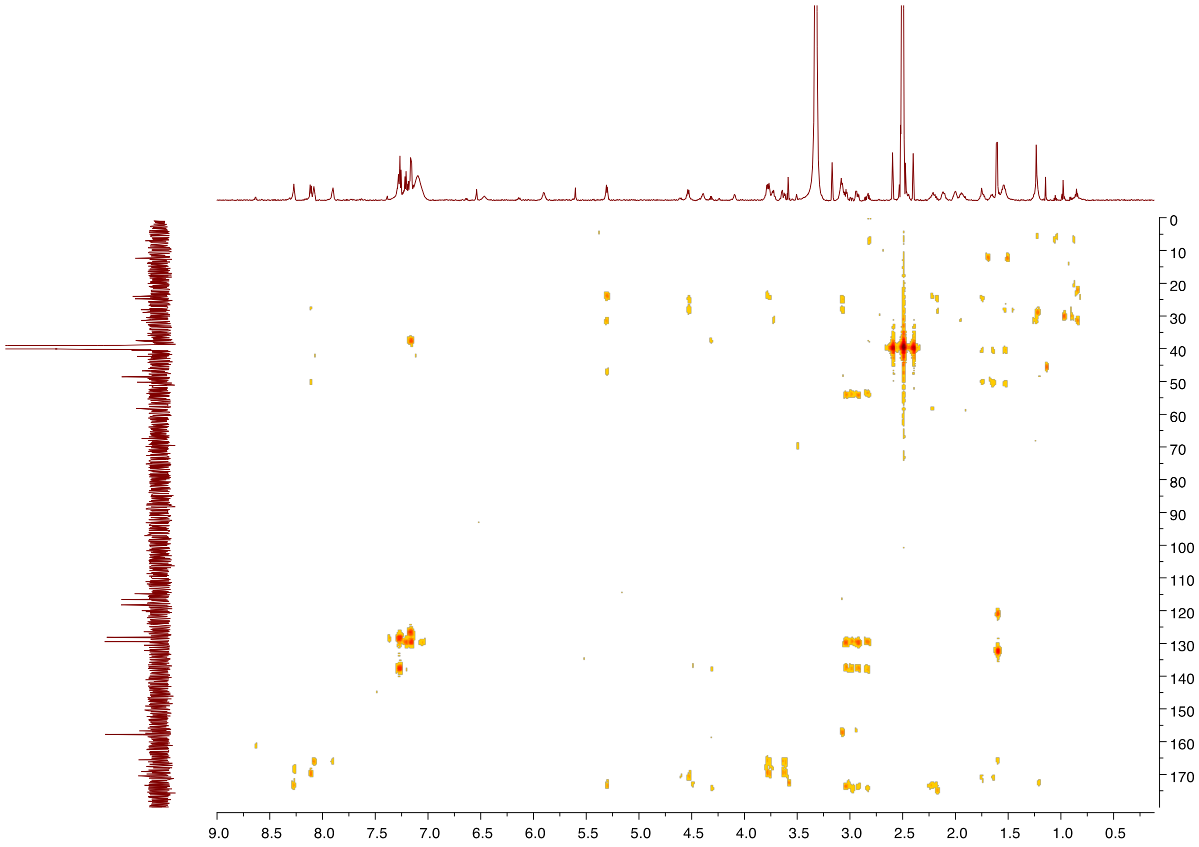

Figure S20. The HMBC spectrum of **6** (700 MHz, DMSO-d_6).

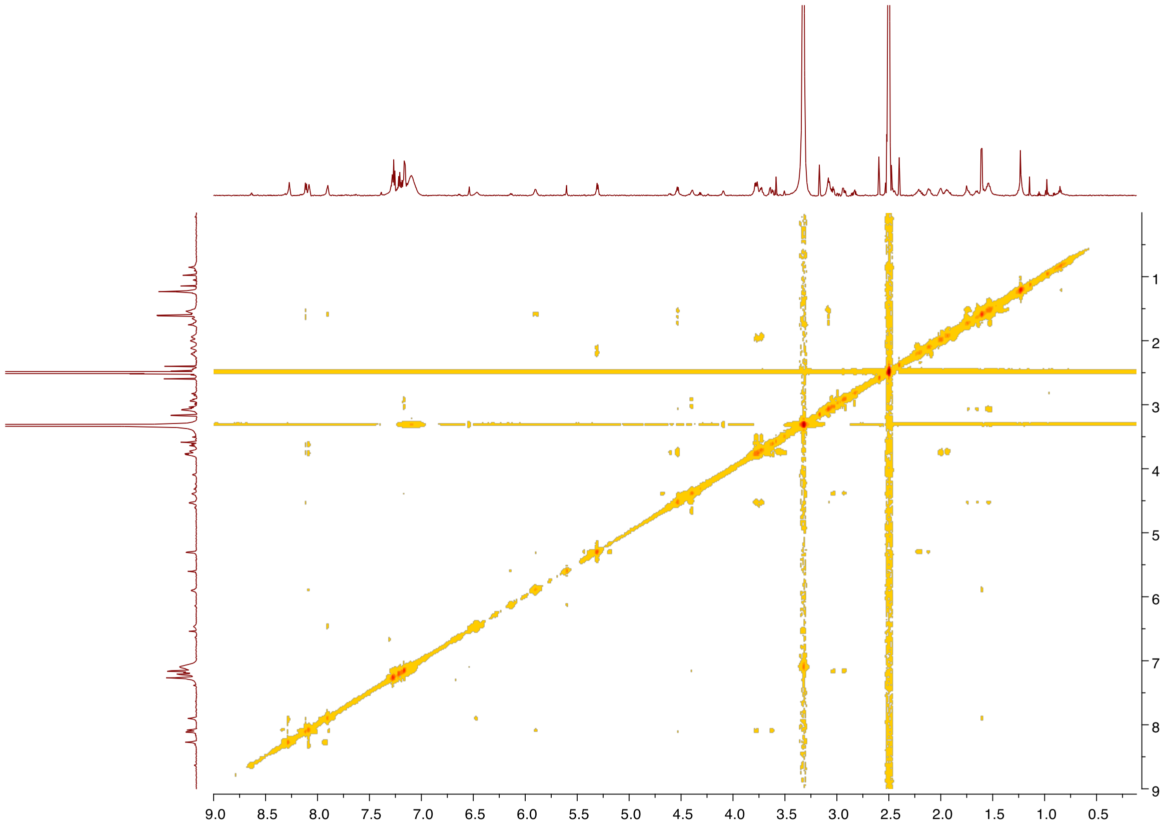

Figure S21. The ROESY spectrum of **6** (700 MHz, DMSO-d_6).

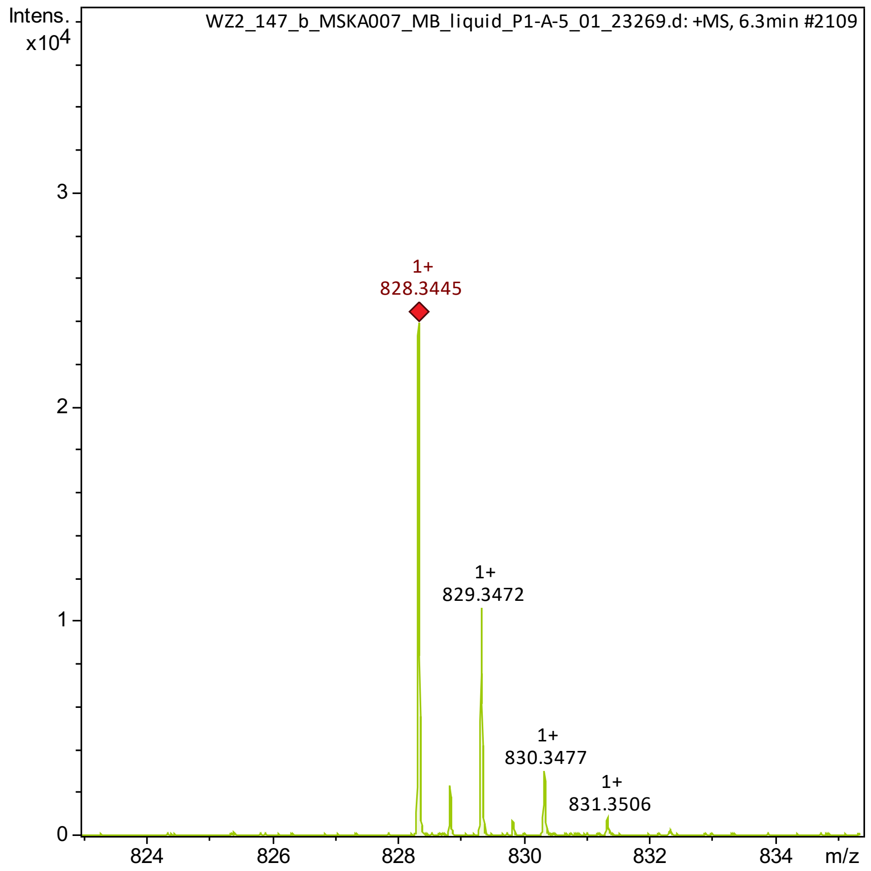

Figure S22. High resolution $[M+H]^{1+}$ MS^1 spectrum for **7**.

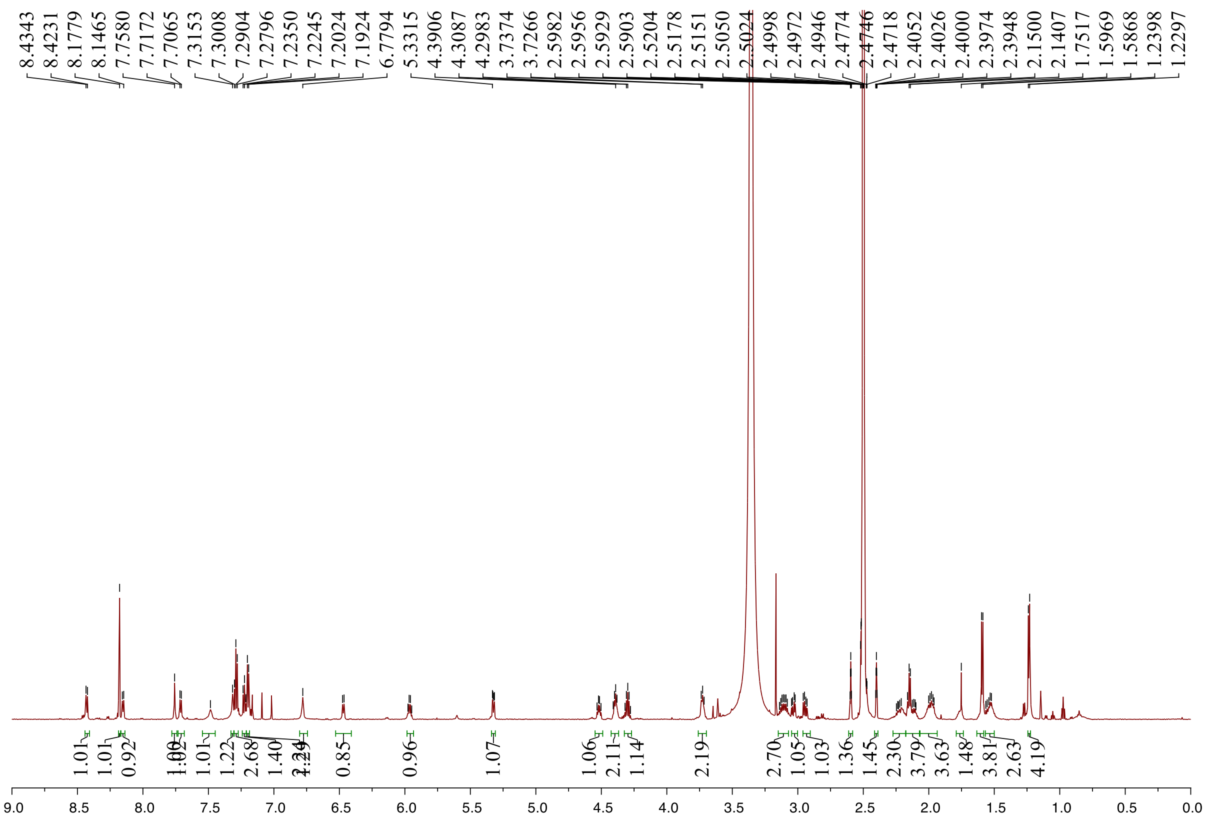

Figure S23. The ^{1}H NMR spectrum of **7** (700 MHz, DMSO-d_6).

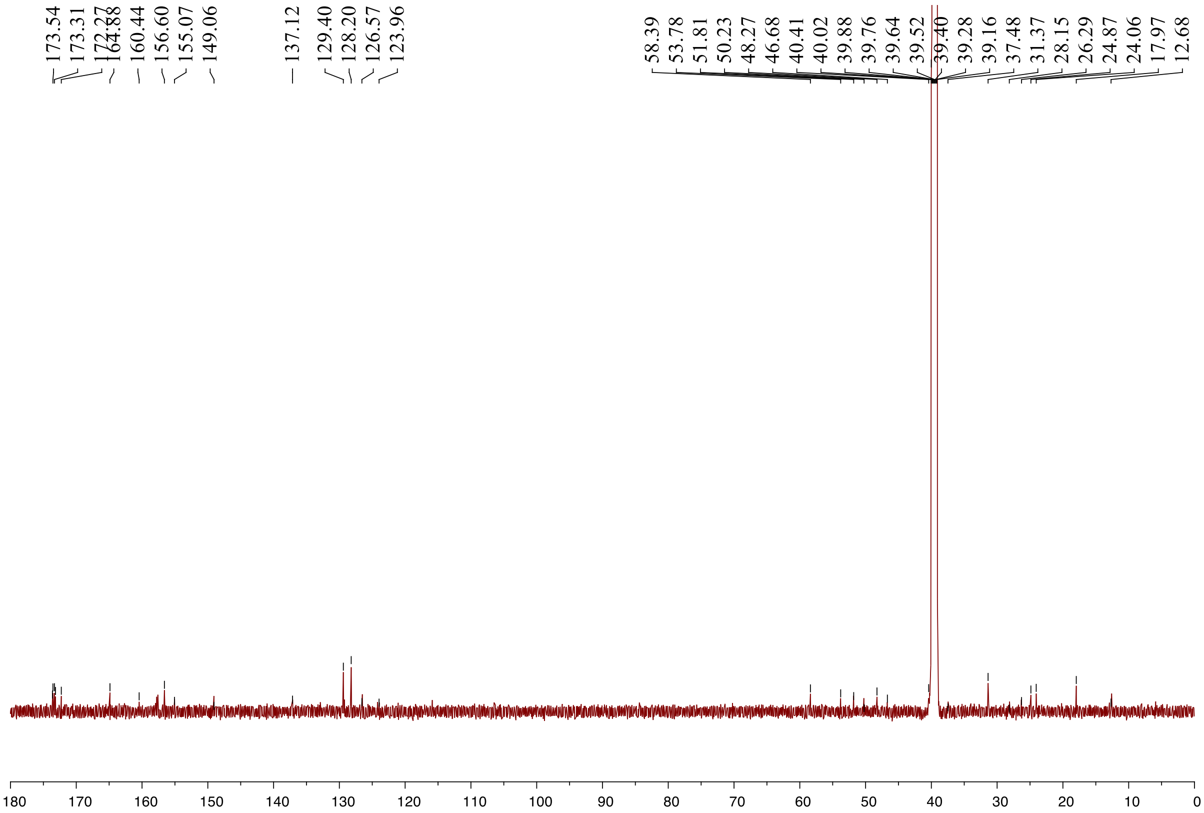

Figure S24. The ^{13}C NMR spectrum of **7** (176 MHz, DMSO-d_6).

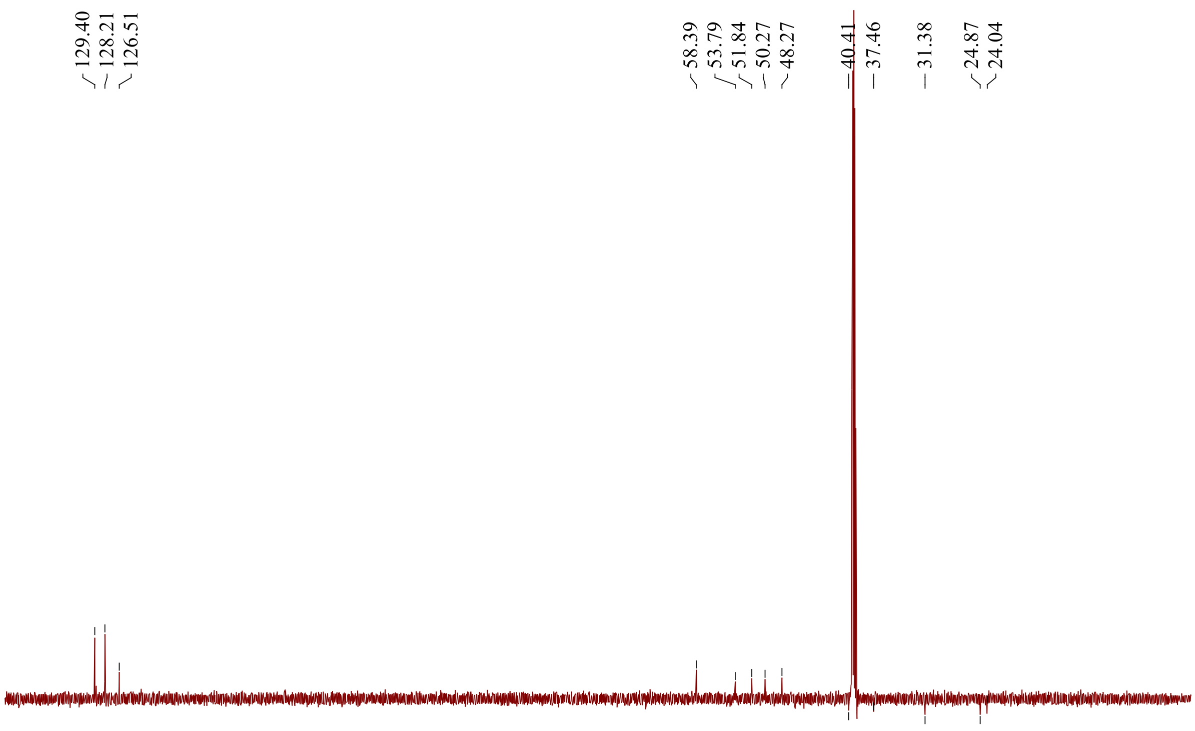

Figure S25. The DEPT135 spectrum of **7** (176 MHz, DMSO-d_6).

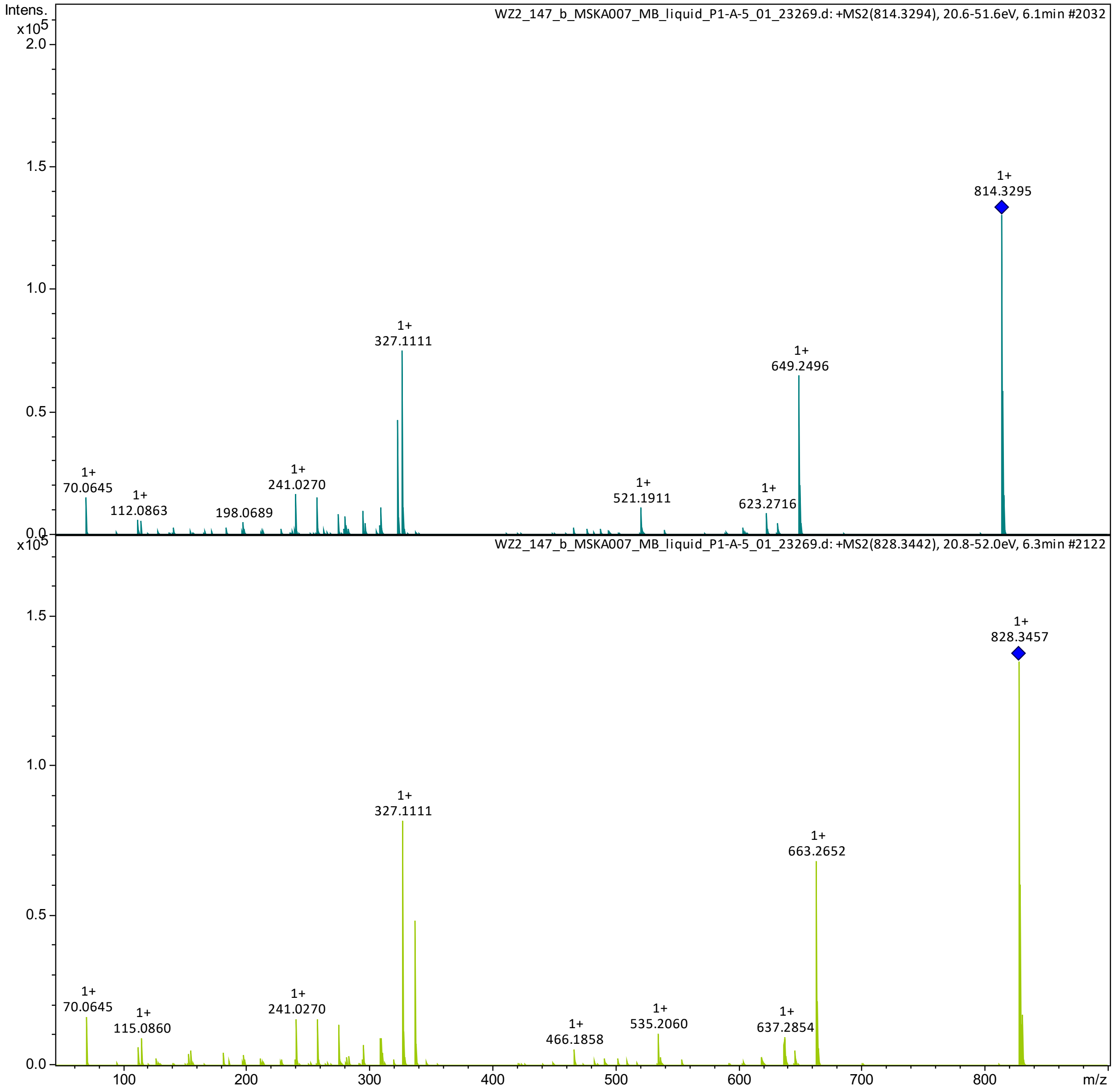

Figure S26. HRMS/MS spectra for **5** (top) and **7** (bottom).

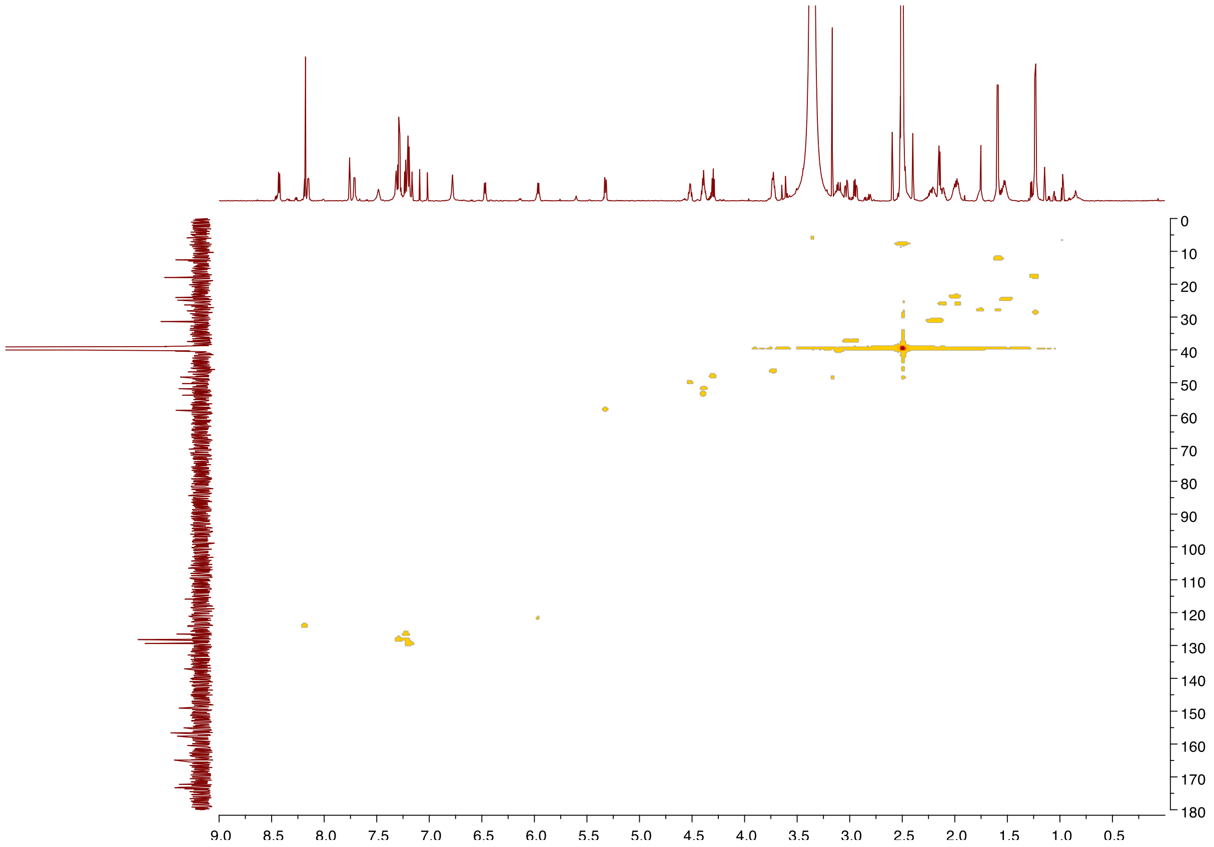

Figure S27. The HSQC spectrum of **7** (700 MHz, DMSO-d_6).

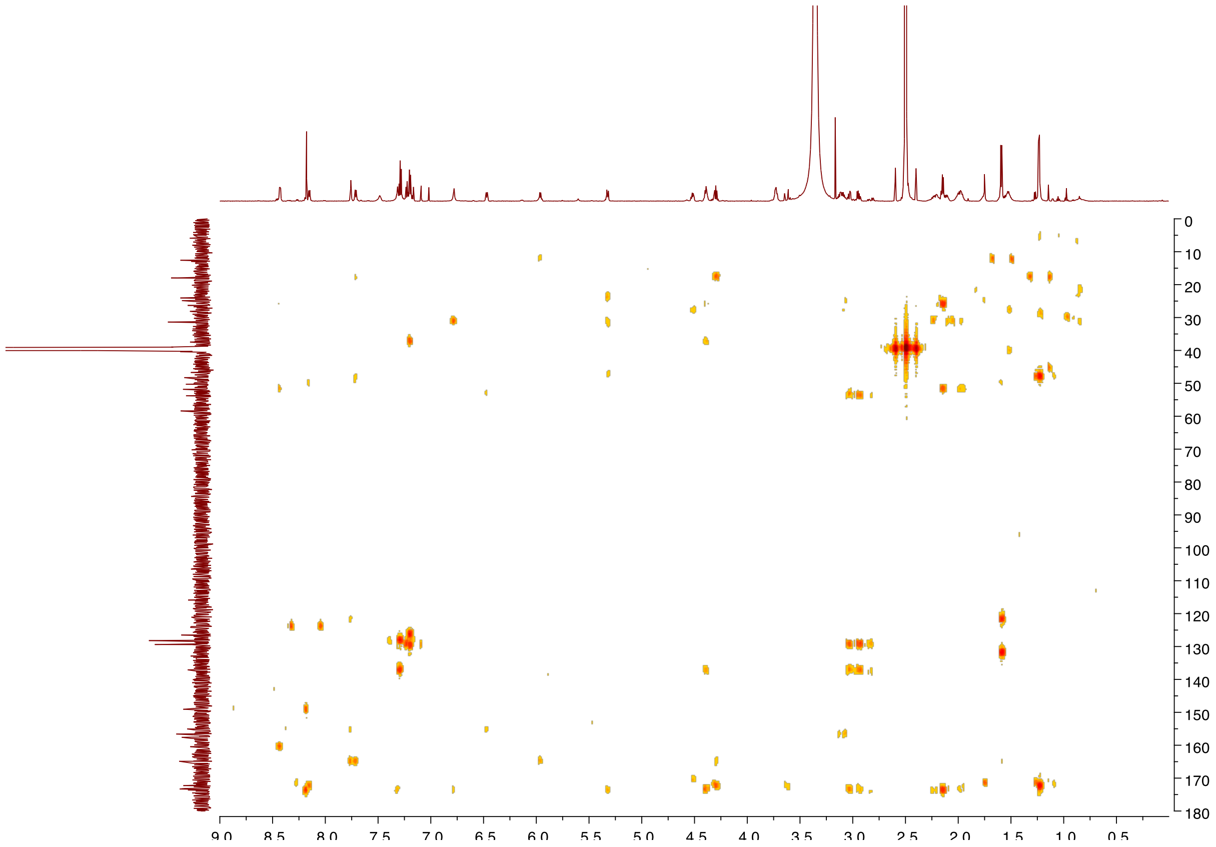

Figure S28. The HMBC spectrum of **7** (700 MHz, DMSO-d_6).

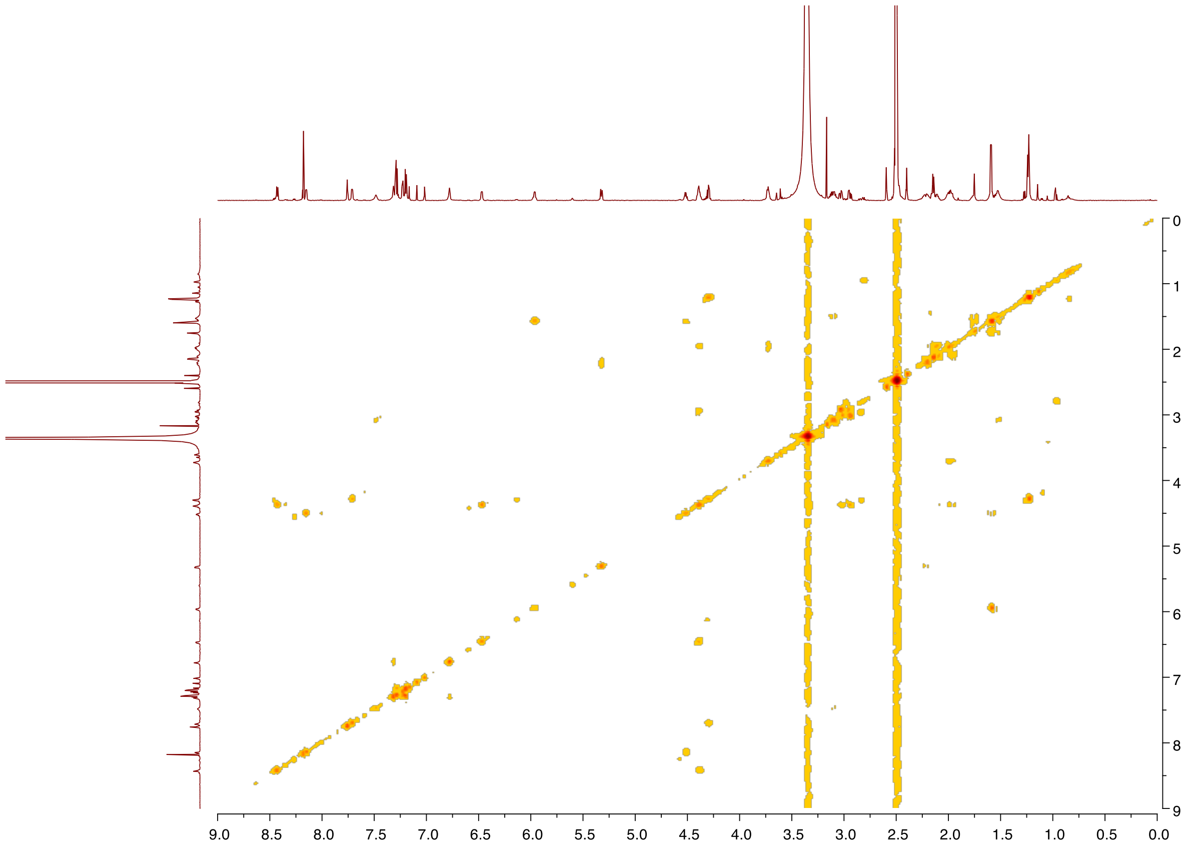

Figure S29. The ^{1}H-^{1}H COSY spectrum of **7** (700 MHz, DMSO-d_6).

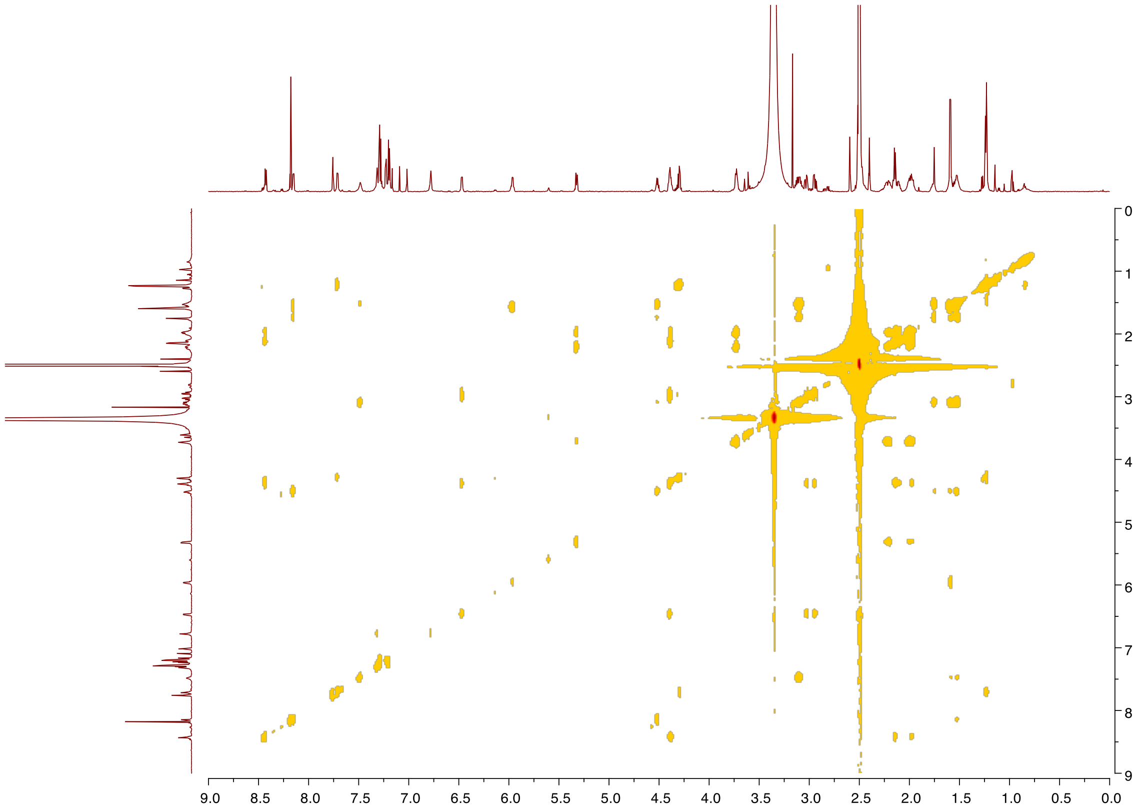

Figure S30. The TOCSY spectrum of **7** (700 MHz, DMSO-d_6).

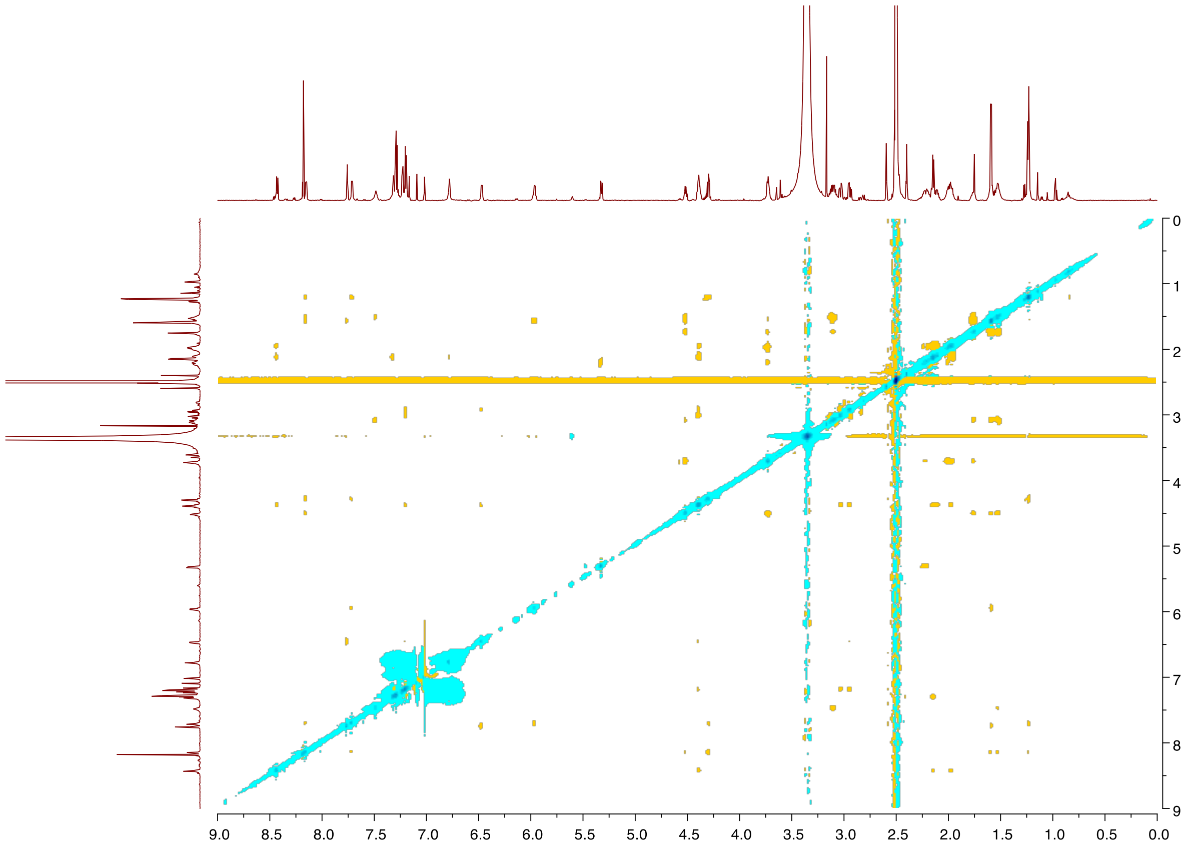

Figure S31. The ROESY spectrum of **7** (700 MHz, DMSO-d_6).

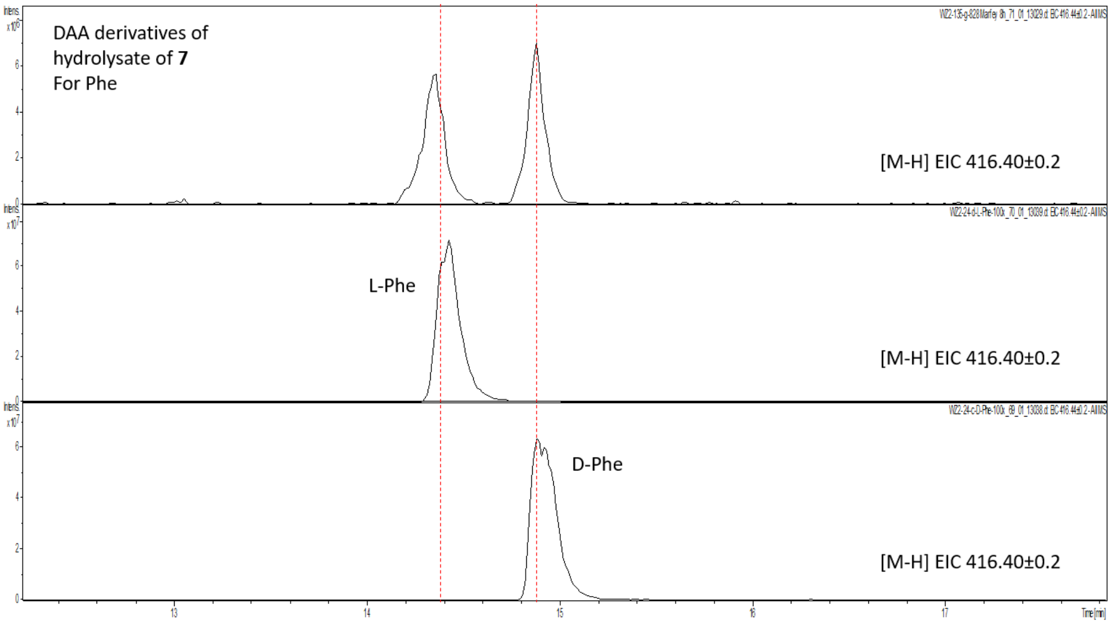

Figure S32. Marfey's analysis to determine the absolute configuration of the Phe residue in **7**. Extracted ion chromatograms (EICs) demonstrating the retention time of the DAA-derivatized Phe residue resulting from the acid hydrolysis of **7** (top), retention time of DAA-derivatized standard of L-Phe (middle), and the retention time of the similarly derivatized standard of D-Phe (bottom). Akin to **5**, the racemization of L-Phe in **7** took place.[1] Separation was achieved using the Agilent Poroshell EC-C18 (100×4.6 mm, 2.7 μm) column. Mass spectrometry data were acquired in the negative ionization mode.

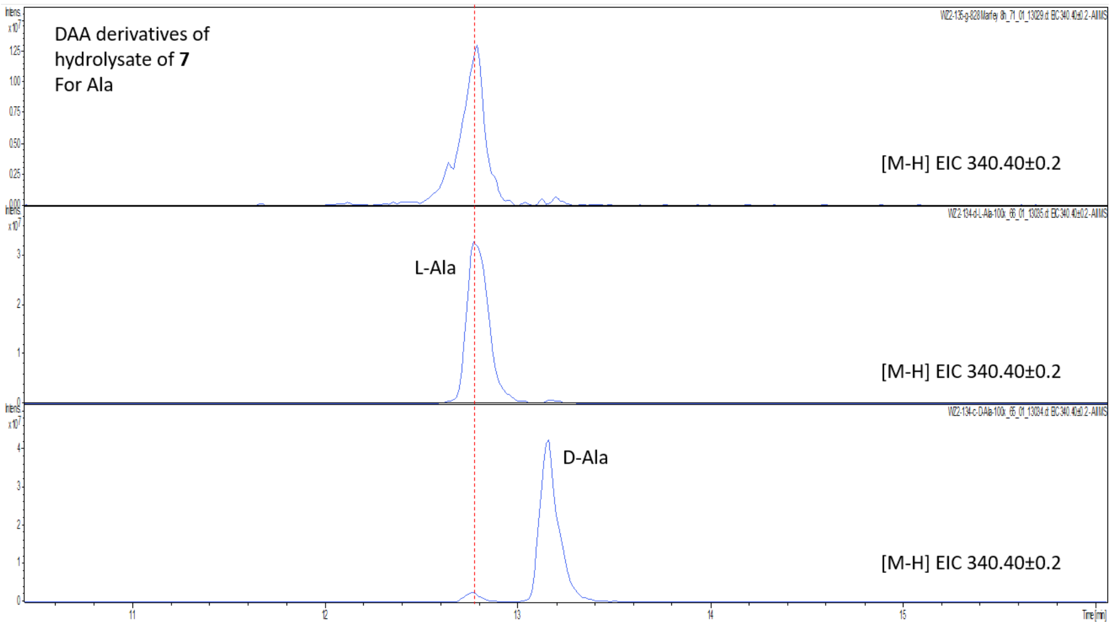

Figure S33. Marfey's analysis to determine the absolute configuration of the Ala residue in **7**. Extracted ion chromatograms (EICs) demonstrating the retention time of the DAA-derivatized Ala residue resulting from the acid hydrolysis of **7** (top), retention time of DAA-derivatized standard of L-Ala (middle), and the retention time of the similarly derivatized standard of D-Ala (bottom). By retention time matching, the Ala residue in **7** was determined to be L-Ala. Separation was achieved using the Agilent Poroshell EC-C18 (100×4.6 mm, 2.7 µm) column. Mass spectrometry data were acquired in the negative ionization mode.

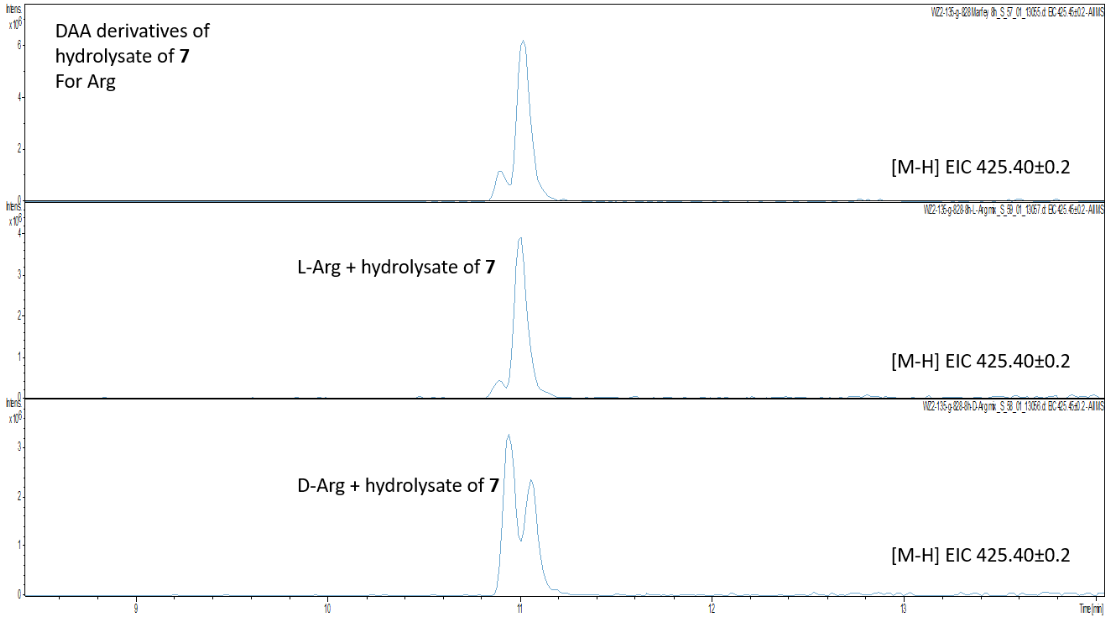

Figure S34. Marfey's analysis to determine the absolute configuration of the Arg residue in **7**. From top to bottom– EICs demonstrating retention time of DAA-derivitized Arg residue obtained by acid hydrolysis of **7**, DAA-derivitized standard for L-Arg spiked with the derivatized acid hydrolysate of **7**, and DAA-derivitized standard for D-Arg spiked with the derivatized acid hydrolysate of **7**. By retention time matching, the Arg residue in **7** was determined to be L-Arg. Separation was achieved using the Agilent Poroshell EC-C18 (100×4.6 mm, 2.7 µm) column. Mass spectrometry data were acquired in the negative ionization mode.

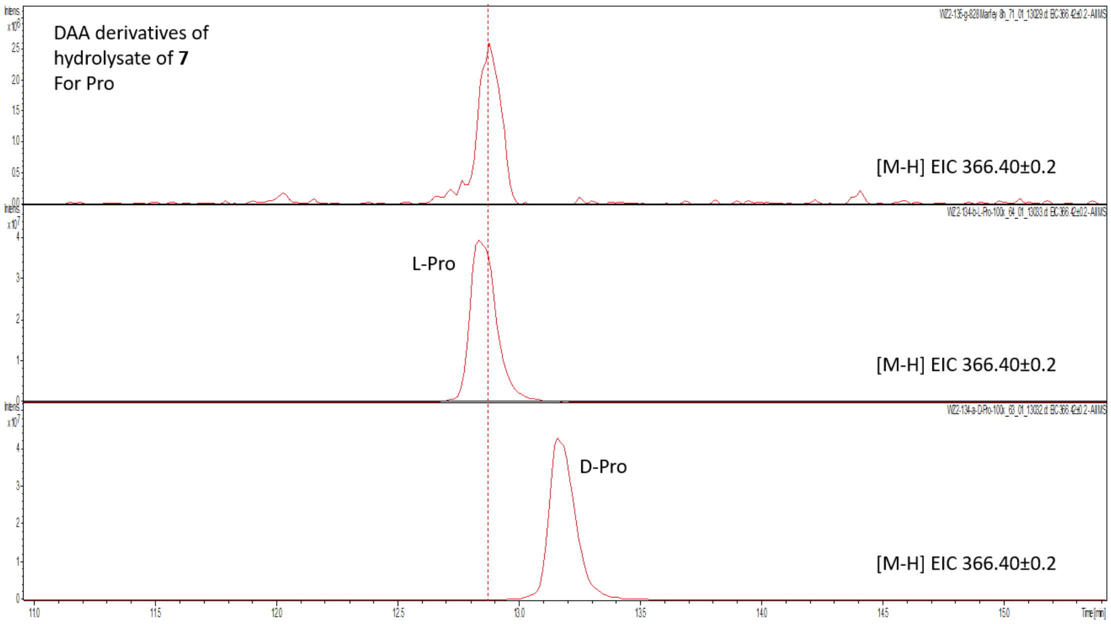

Figure S35. Marfey's analysis to determine the absolute configuration of the Pro residue in **7**. Extracted ion chromatograms (EICs) demonstrating the retention time of the DAA-derivatized Pro residue resulting from the acid hydrolysis of **7** (top), retention time of DAA-derivatized standard of L-Pro (middle), and the retention time of the similarly derivatized standard of D-Pro (bottom). By retention time matching, the Pro residue in **7** was determined to be L-Pro. Separation was achieved using the Agilent Poroshell EC-C18 (100×4.6 mm, 2.7 µm) column. Mass spectrometry data were acquired in the negative ionization mode.

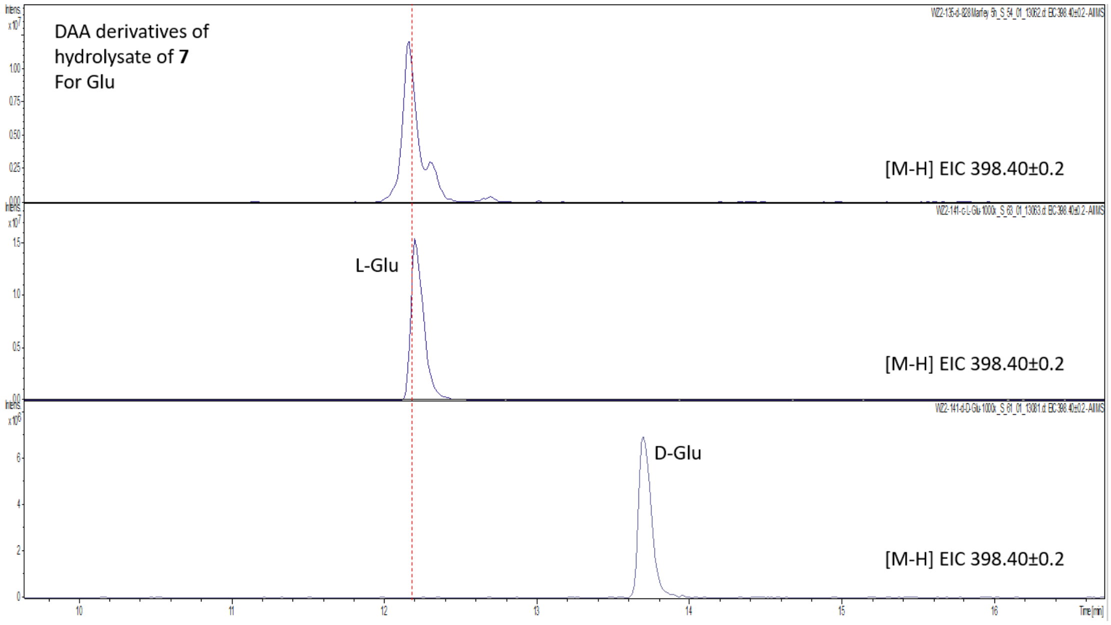

Figure S36. Marfey's analysis to determine the absolute configuration of the Gln residue in **7**. During acid hydrolysis, Gln is converted to Glu; hence, Glu standards are used here. Extracted ion chromatograms (EICs) demonstrating the retention time of the DAA-derivatized Glu residue resulting from the acid hydrolysis of **7** (top), retention time of DAA-derivatized standard of L-Glu (middle), and the retention time of the similarly derivatized standard of D-Glu (bottom). By retention time matching, the Gln residue in **7** was determined to be L-Gln. Separation was achieved using the Agilent Poroshell EC-C18 (100×4.6 mm, 2.7 μm) column. Mass spectrometry data were acquired in the negative ionization mode.

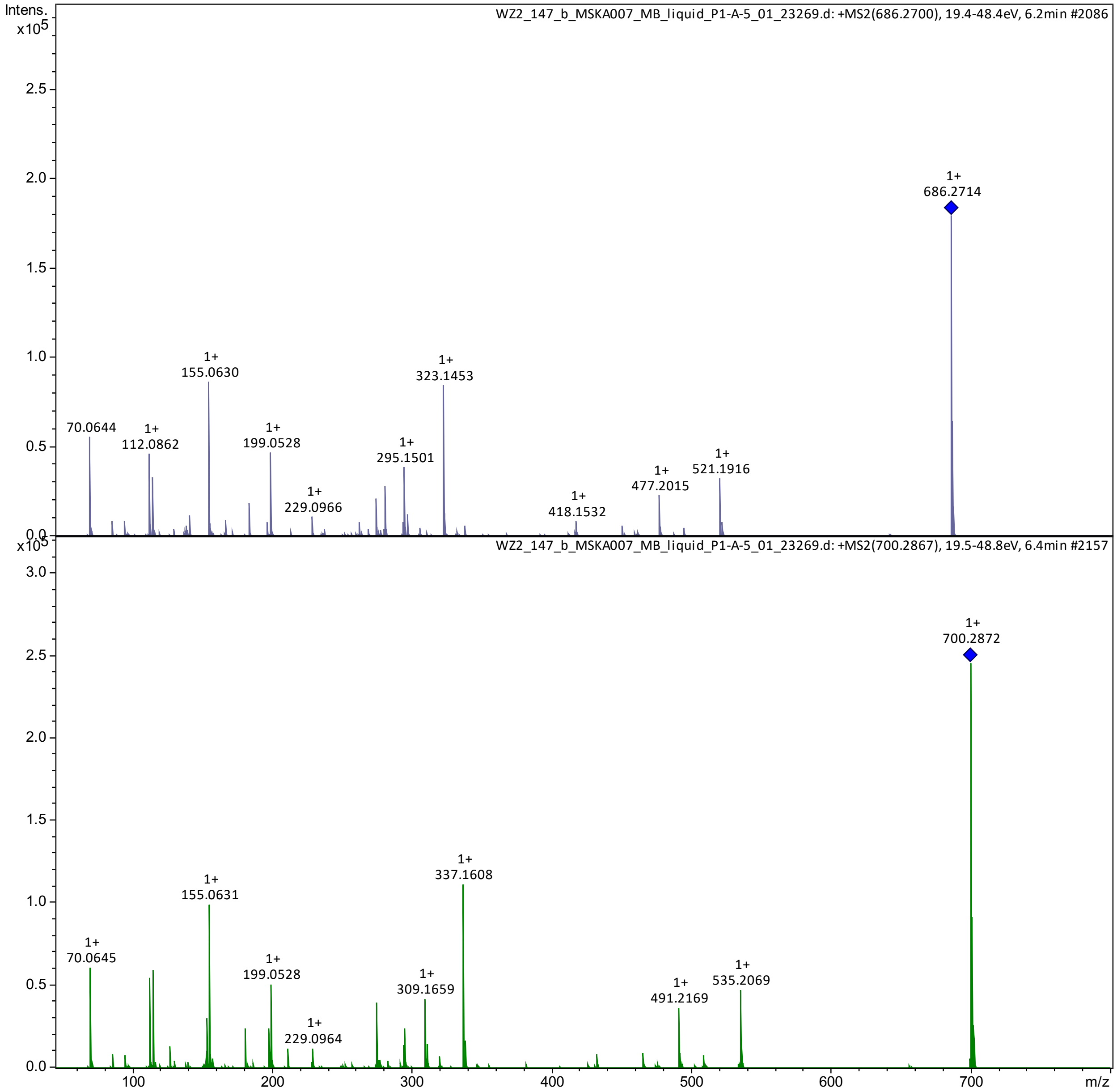

Figure S37. HRMS/MS spectra for **6** (top) and **8** (bottom).

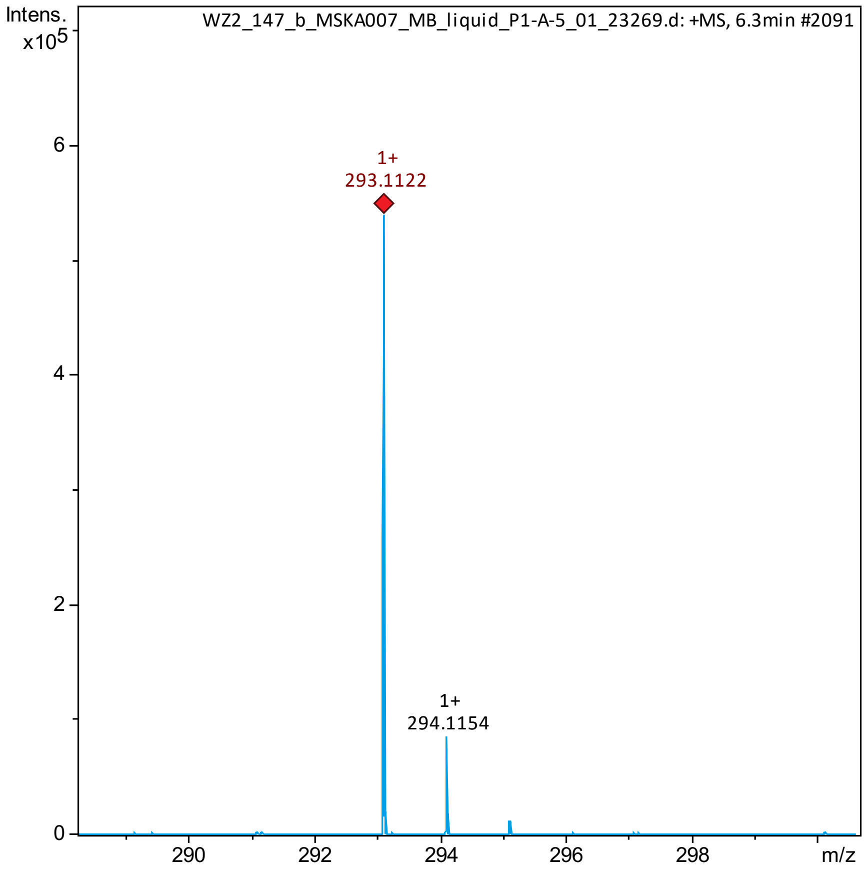

Figure S38. High resolution $[M+H]^{1+}$ MS^1 spectrum for **9**.

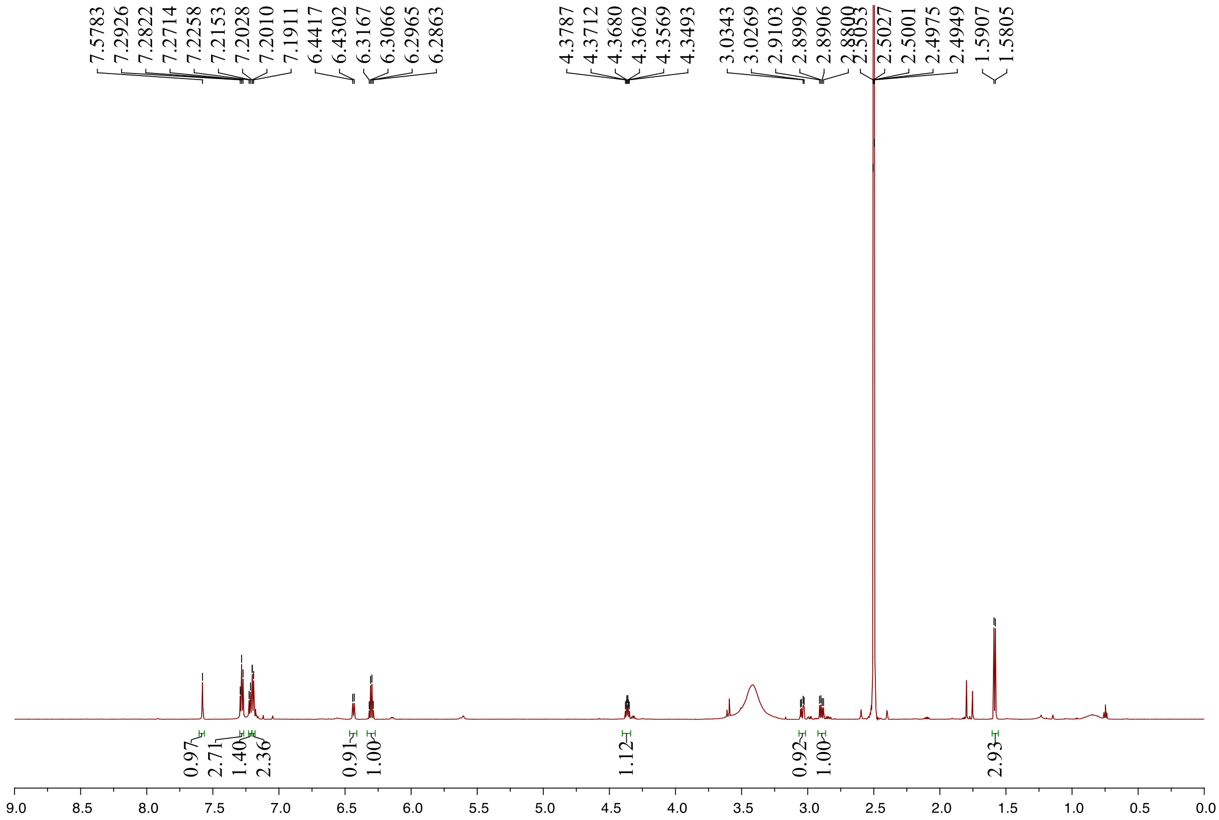

Figure S39. The ^{1}H NMR spectrum of **9** (700 MHz, DMSO-d_6).

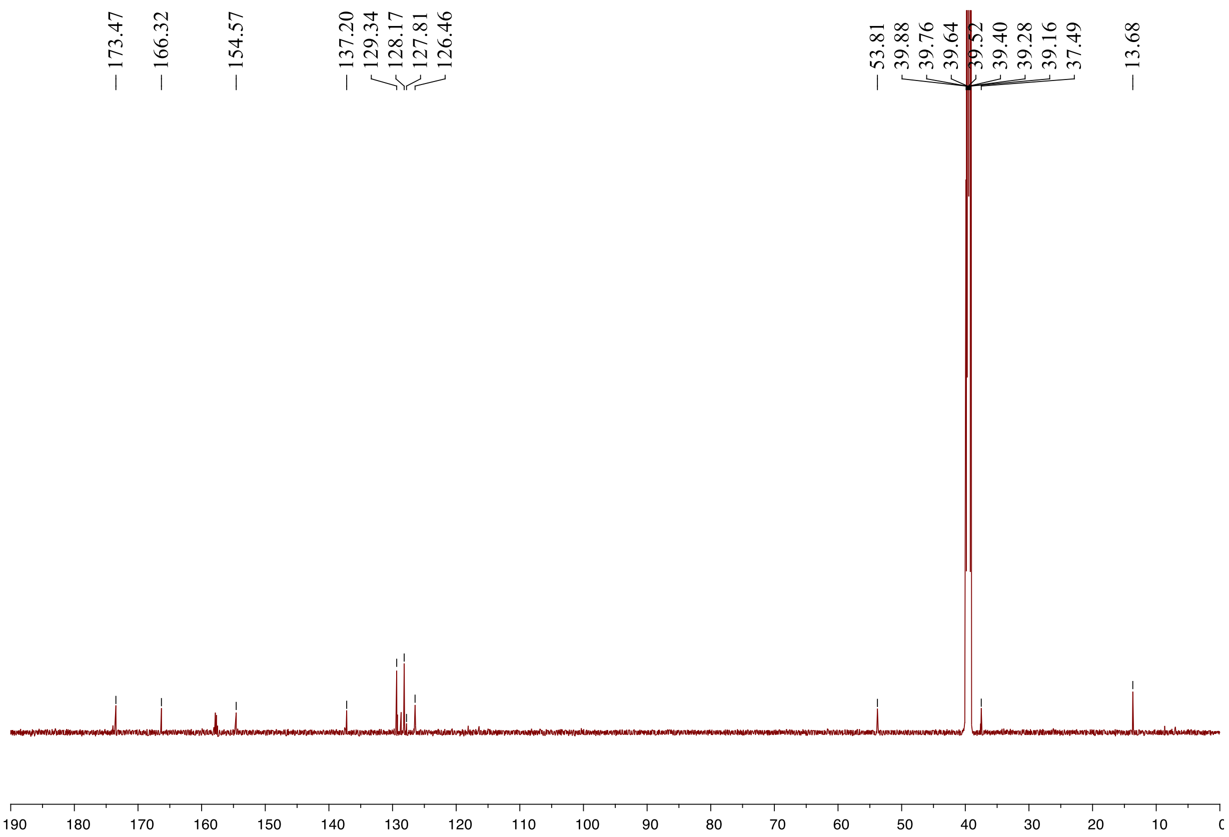

Figure S40. The ^{13}C NMR spectrum of **9** (176 MHz, DMSO-d_6).

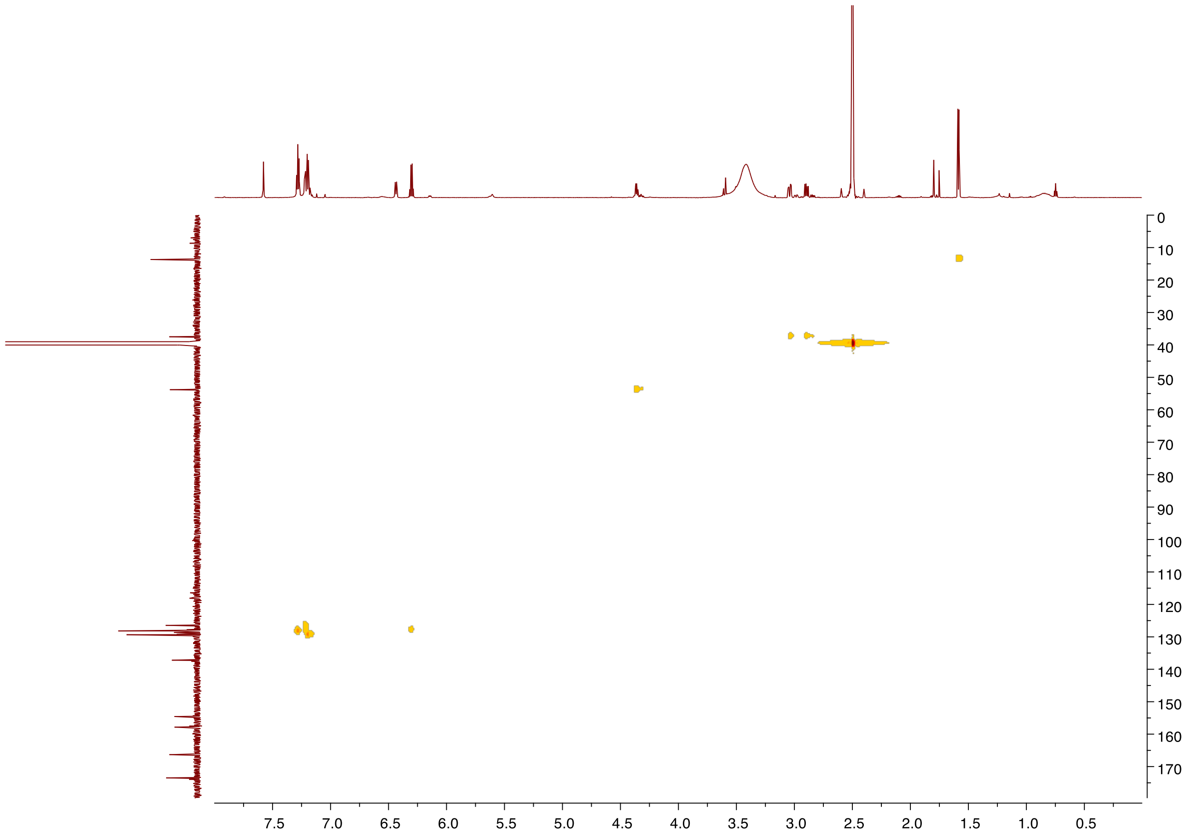

Figure S41. The HSQC spectrum of **9** (700 MHz, DMSO-d_6).

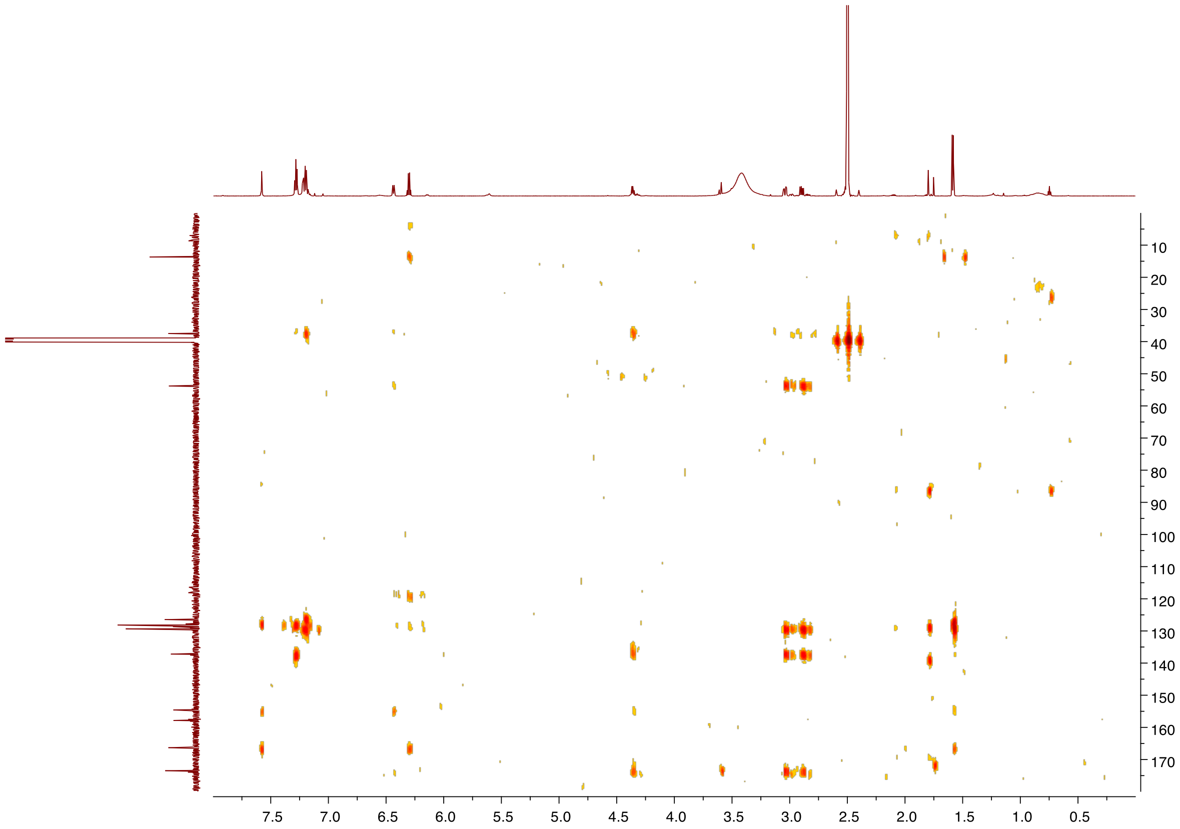

Figure S42. The HMBC spectrum of **9** (700 MHz, DMSO-d_6).

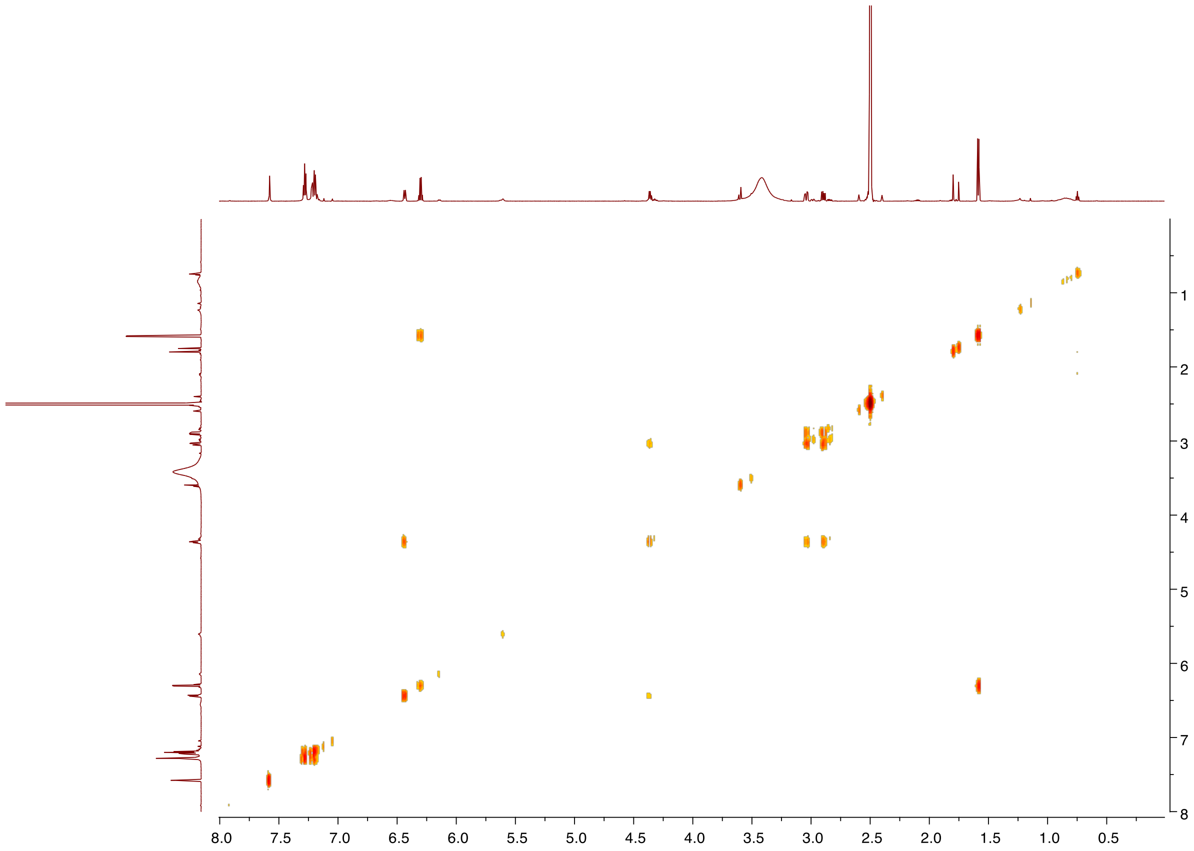

Figure S43. The ^{1}H-^{1}H COSY spectrum of **9** (700 MHz, DMSO-d_6).

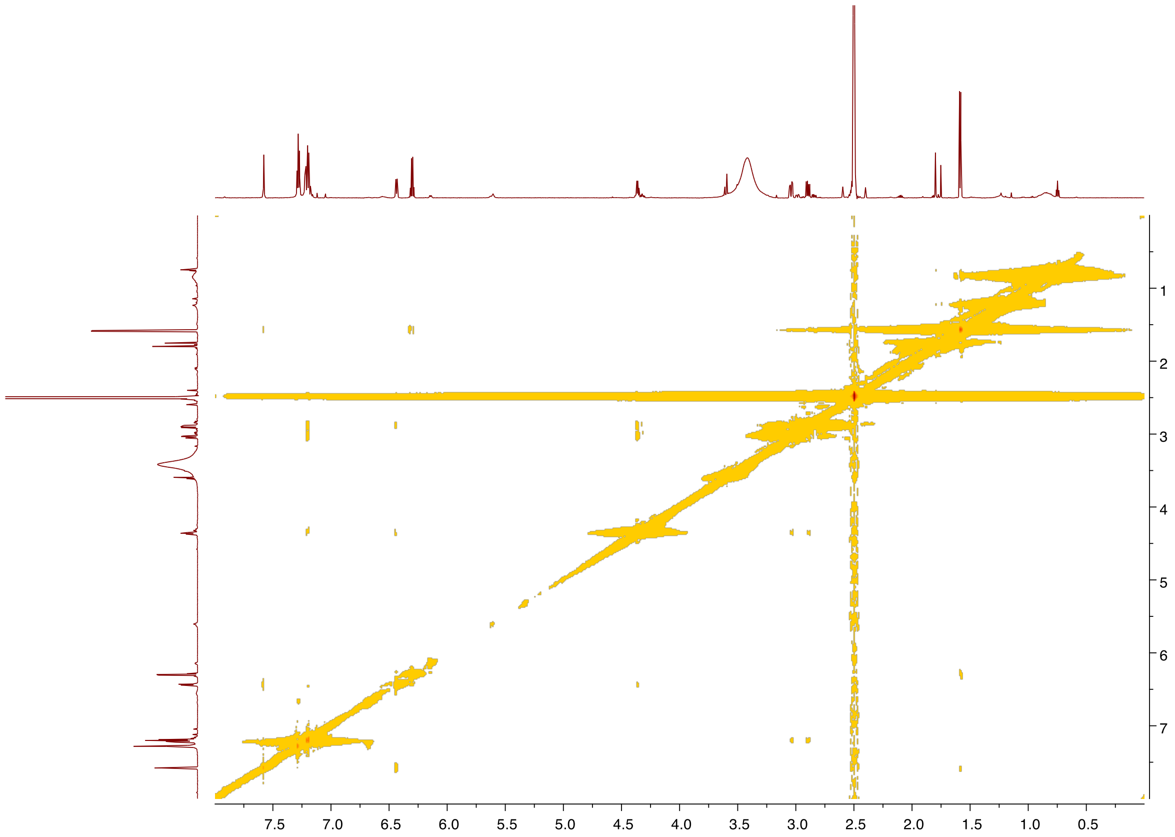

Figure S44. The ROESY spectrum of **9** (700 MHz, DMSO-d_6).

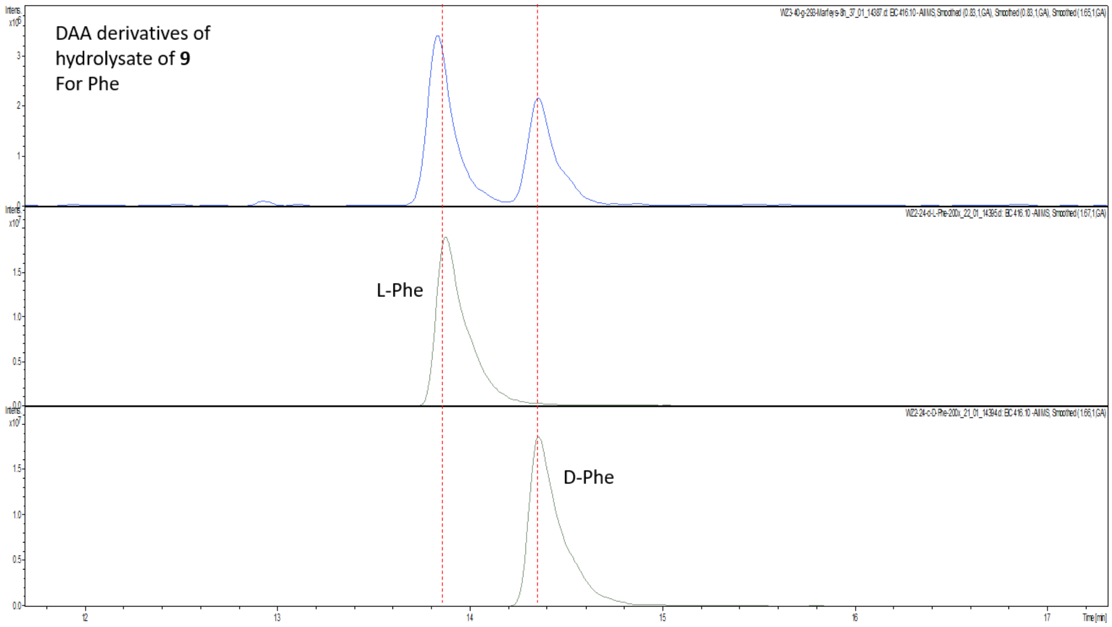

Figure S45. Marfey's analysis to determine the absolute configuration of the Phe residue in **9**. Extracted ion chromatograms (EICs) demonstrating the retention time of the DAA-derivatized Phe residue resulting from the acid hydrolysis of **9** (top), retention time of DAA-derivatized standard of L-Phe (middle), and the retention time of the DAA-derivatized standard of D-Phe (bottom). Akin to **5** and **7**, the racemization of L-Phe in **9** took place.[1] Separation was achieved using the Agilent Poroshell EC-C18 (100×4.6 mm, 2.7 μm) column. Mass spectrometry data were acquired in the negative ionization mode.

SUPPLEMENTARY REFERENCES

(1) Kanki, D.; Nakamukai, S.; Ogura, Y.; Takikawa, H.; Ise, Y.; Morii, Y.; Yamawaki, N.; Takatani, T.; Arakawa, O.; Okada, S.; Matsunaga, S. *J Nat Prod* **2021**, *84*, 1848.
